# Blockade of the immunosuppressive KIR2DL5/PVR pathway elicits potent human NK cell–mediated antitumor immunity

**DOI:** 10.1172/JCI163620

**Published:** 2022-11-15

**Authors:** Xiaoxin Ren, Mou Peng, Peng Xing, Yao Wei, Phillip M. Galbo, Devin Corrigan, Hao Wang, Yingzhen Su, Xiaoshen Dong, Qizhe Sun, Yixian Li, Xiaoyu Zhang, Winfried Edelmann, Deyou Zheng, Xingxing Zang

**Affiliations:** 1 Department of Microbiology and Immunology and; 2 Department of Genetics, Albert Einstein College of Medicine, Bronx, New York, USA.; 3 Division of Pediatric Hematology/Oncology/Transplant and Cellular Therapy, Children’s Hospital at Montefiore, Bronx, New York, USA.; 4 Department of Cell Biology,; 5 Departments of Neurology and Neuroscience,; 6 Department of Oncology,; 7 Department of Medicine, and; 8 Department of Urology, Albert Einstein College of Medicine, Bronx, New York, USA.

**Keywords:** Immunology, Oncology, Cancer immunotherapy

## Abstract

Cancer immunotherapy targeting the TIGIT/PVR pathway is currently facing challenges. KIR2DL5, a member of the human killer cell, immunoglobulin-like receptor (KIR) family, has recently been identified as another binding partner for PVR. The biology and therapeutic potential of the KIR2DL5/PVR pathway are largely unknown. Here we report that KIR2DL5 was predominantly expressed on human NK cells with mature phenotype and cytolytic function and that it bound to PVR without competition with the other 3 known PVR receptors. The interaction between KIR2DL5 on NK cells and PVR on target cells induced inhibitory synapse formation, whereas new monoclonal antibodies blocking the KIR2DL5-PVR interaction robustly augmented the NK cytotoxicity against PVR^+^ human tumors. Mechanistically, both intracellular ITIM and ITSM of KIR2DL5 underwent tyrosine phosphorylation after engagement, which was essential for KIR2DL5-mediated NK suppression by recruiting SHP-1 and/or SHP-2. Subsequently, ITIM/SHP-1/SHP-2 and ITSM/SHP-1 downregulated the downstream Vav1/ERK1/2/p90RSK/NF-κB signaling. KIR2DL5^+^ immune cells infiltrated in various types of PVR^+^ human cancers. Markedly, the KIR2DL5 blockade reduced tumor growth and improved overall survival across multiple NK cell–based humanized tumor models. Thus, our results revealed functional mechanisms of KIR2DL5-mediated NK cell immune evasion, demonstrated blockade of the KIR2DL5/PVR axis as a therapy for human cancers, and provided an underlying mechanism for the clinical failure of anti-TIGIT therapies.

## Graphical abstract

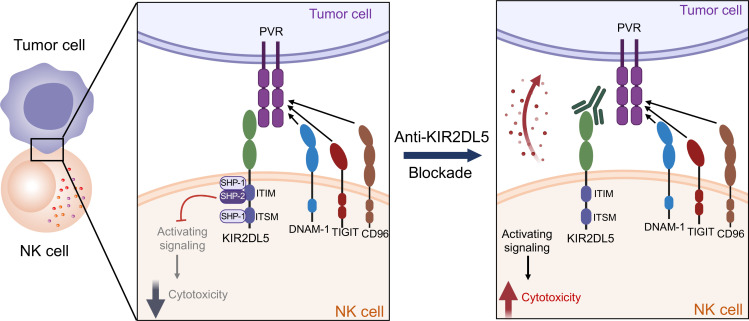

## Introduction

Cancer immunotherapy has made great progress due to immune checkpoint inhibitors targeting cytotoxic T lymphocyte–associated antigen 4 (CTLA-4) and programmed cell death protein 1/programmed death ligand 1 (PD-1/PD-L1) in the clinic (1–3). Despite the success of the checkpoint inhibitors in improving clinical outcomes, only a small subset of patients (~10%–30%) exhibit durable and long-term responses (3, 4). The immunological effects of these immunotherapies are limited to T cells owing to their specific expression profiles. Natural killer (NK) cells are also crucial effector cells in immunosurveillance against infected and transformed cells (5). Consequently, NK cell–based cancer immunotherapy, such as chimeric antigen receptor NK and cytokine-induced memory-like NK cells, has emerged as another treatment approach being tested clinically (6–8). The functionality of NK cells is manipulated by a wide range of activating and inhibitory receptors that recognize their respective ligands on target cells or antigen-presenting cells (9–11). Deficient and dysfunctional NK cells have been associated with an increased incidence rate and enhanced growth and metastasis in various cancers, leading to poor clinical outcomes (12). Emerging immune checkpoint molecules have been found to mediate NK cell dysfunction in the tumor microenvironment (TME) (13, 14); thus exploring the therapeutic potential of NK cell–based immune checkpoints is particularly interesting.

Human killer cell, immunoglobulin-like receptors (KIRs) regulate NK activity by recognizing self–HLA class I molecules, which enables NK cells to kill infected and tumor cells through the “missing self” mechanism (15, 16). Among these KIRs that are less well defined, KIR2DL5 is the most recently identified. Unlike KIR2DL1–3, which recognize HLA-C through extracellular Ig-like D1–D2 domains (17, 18), KIR2DL5 displays an Ig-like D0–D2 configuration and constitutes an ancestral lineage of KIR with KIR2DL4, which is an HLA-G receptor (19). KIR2DL5 is encoded by 2 paralogous genes, *KIR2DL5A* and *KIR2DL5B*, displaying highly allelic polymorphism like other KIRs (20, 21). While most *KIR2DL5B* alleles are transcriptionally silent because of an impaired RUNX binding site conserved in the promoter region of most KIRs, all known *KIR2DL5A* alleles and a few *KIR2DL5B* alleles have intact RUNX binding sites and are expected to be expressed (22). KIR2DL5 was considered as an orphan molecule until very recent studies identified it as a new binding partner of poliovirus receptor (PVR, also known as CD155) using high-throughput screening of receptor-ligand interactions (23–25). As a newly identified pathway, the biology of KIR2DL5/PVR is largely unknown.

PVR is a member of the nectin/nectin-like family, which mediates cell adhesion, invasion and migration, and proliferation (26). Expression of PVR is low or absent in most healthy tissues; however, it is overexpressed on numerous types of tumors, including colorectal cancer, glioma, myeloid leukemia, ovarian cancer, lung cancer, pancreatic cancer, melanoma, and other tumors (27). Accumulating evidence suggests that PVR overexpression induces the immune escape of tumor cells and is associated with a poor prognosis and enhanced tumor progression (28–31). Besides its tumor-intrinsic roles, PVR participates in multiple immunoregulatory events through finely tuned interaction with the stimulatory receptor DNAX accessory molecule 1 (DNAM-1, also known as CD226) and the inhibitory receptors T cell immunoreceptor with Ig and ITIM domains (TIGIT) and CD96 (32–34). Immunotherapies targeting the TIGIT/PVR axis are now being actively explored in clinical trials in various cancer patients (35–40). However, the role of the KIR2DL5/PVR pathway in TME and the therapeutic potential targeting this pathway have not been explored yet.

In the present study, we developed new specific mAbs that recognized KIR2DL5 more efficiently than the commercial mAb UP-R1 and determined the predominant expression of KIR2DL5 on NK cells with mature phenotype and cytolytic function. We further defined the features of KIR2DL5 as a receptor for PVR and found that KIR2DL5 and the other known PVR receptors DNAM-1, TIGIT, and CD96 bound nonidentical sites on PVR. Our results demonstrated that KIR2DL5 functioned as an inhibitory receptor on NK cells and mediated PVR^+^ tumor immune resistance. Mechanistically, both immunoreceptor tyrosine-based inhibitory motif (ITIM) and immunoreceptor tyrosine-based switch motif (ITSM) in the KIR2DL5 cytoplasmic domain were essential for KIR2DL5-mediated NK cell inhibition through recruiting Src homology 2–containing protein tyrosine phosphatase 1 (SHP-1) and SHP-2, and therefore downregulated downstream Vav guanine nucleotide exchange factor 1 (Vav1), extracellular signal–regulated kinase 1/-2 (ERK1/2), p90 ribosomal S6 kinase (p90RSK), and NF-κB signaling. Moreover, we observed KIR2DL5^+^ immune cells infiltrated in various PVR^+^ human cancers. Finally, multiple humanized tumor models demonstrated that blockade of KIR2DL5 prevented NK cell dysfunction and promoted NK cell–mediated antitumor activity. Our findings set the cellular and molecular basis for the inhibitory function of KIR2DL5 and demonstrate the therapeutic potential of blocking the KIR2DL5/PVR pathway in NK cell–based cancer immunotherapy. Our results also provide an underlying mechanism for recent clinical failure of anti-TIGIT therapies.

## Results

### Generation and characterization of new anti-KIR2DL5 specific mAbs.

**Figure 1:**
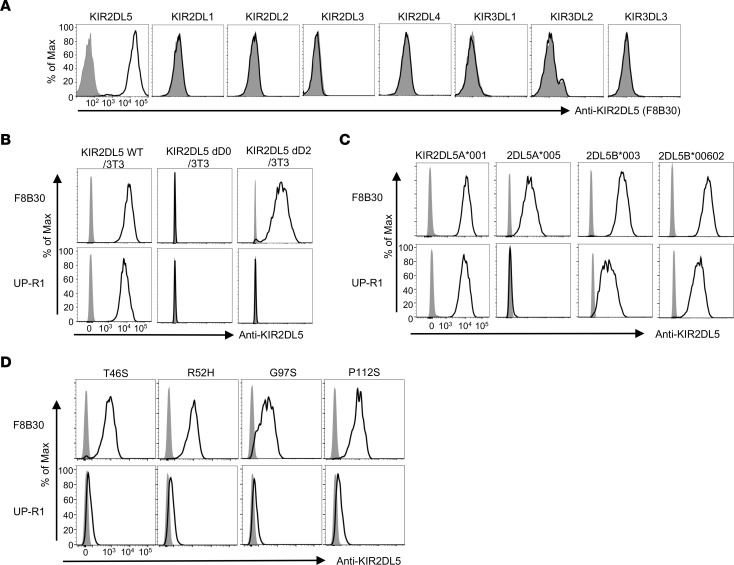
Generation and characterization of anti-KIR2DL5–specific mAbs. (**A**) The specificity of anti-KIR2DL5 mAb clone F8B30. 3T3 cells transduced with indicated KIR family members were stained with 5 μg/mL of F8B30 (open) or mIgG1 (shaded). (**B**) 3T3 cells transduced with D0-deleted (KIR2DL5 dD0) or D2-deleted KIR2DL5 (KIR2DL5 dD2) were stained with 5 μg/mL of clone F8B30 or commercial clone UP-R1. (**C**) Anti-KIR2DL5 mAb clone F8B30 recognized different KIR2DL5A and 5B alleles. 3T3 cells transduced with indicated alleles were stained with 5 μg/mL of F8B30 or UP-R1 (open) or mIgG1 (shaded). (**D**) Anti-KIR2DL5 mAb clone F8B30 recognized different KIR2DL5 D0 domain variants. 3T3 cells transduced with indicated D0 variants were stained with 0.025 μg/mL of F8B30 or UP-R1 (open) or mIgG1 (shaded). In **A**–**D**, data are representative of 2 independent experiments.

KIR2DL5 is a type I transmembrane glycoprotein characterized by 2 extracellular Ig-like D0–D2 domains, a transmembrane domain, and an intracellular tail (Supplemental Figure 1A; supplemental material available online with this article; https://doi.org/10.1172/JCI163620DS1). Development of the anti-KIR2DL5 mAb UP-R1 enables specific KIR2DL5 detection (41). However, not every *KIR2DL5*^+^ individual is detectable by UP-R1 (20, 24). To further define the expression pattern of KIR2DL5, we generated 8 new anti-KIR2DL5 specific mAbs, which had no cross-reaction with other KIRs (Figure 1A and Supplemental Figure 1B). Our lead clone F8B30 displayed high affinity against KIR2DL5 (*K_D_* = 0.72 nM) as determined by biolayer interferometry (Supplemental Figure 1, C and D). To determine the KIR2DL5 recognition pattern of these new mAbs, we expressed 2 truncated KIR2DL5 proteins by removing the D0 or D2 domain. In comparison with UP-R1, which required both D0 and D2 domains for KIR2DL5 recognition, several of our anti-KIR2DL5 mAbs, including F8B30, bound to KIR2DL5 through the D0 domain (Figure 1B and Supplemental Figure 1E).

Like other KIRs, KIR2DL5 is highly polymorphic (42). KIR2DL5A is most represented by 2DL5A*001, against which our mAbs were generated. 2DL5A*005 is the second most common KIR2DL5A allele and is weakly expressed on the cell surface (20). Notably, F8B30, but not UP-R1, efficiently recognized cell surface–expressed 2DL5A*005 (Figure 1C). While most KIR2DL5B alleles are epigenetically silent because of a distinctive substitution in a promoter RUNX binding site, 2DL5B*003 and 2DL5B*00602 alleles with intact RUNX binding sites are predicted to be transcribed and expressed on the cell surface (22). These 2 alleles have an identical D0 domain to KIR2DL5A*001 and thus are hypothesized to be recognized by our mAbs. As expected, 2DL5B*003 and 2DL5B*00602 could also be bound by F8B30 and other clones (Figure 1C and Supplemental Figure 2A).

KIR2DL5 D0 domain, through which F8B30 recognized KIR2DL5, contains only 4 polymorphic sites: T46S, R52H, G97S, and P112S (IPD-KIR Database, Release 2.9.0) (43). To examine whether D0 domain polymorphism affects KIR2DL5 recognition by our mAbs, we generated these four D0 variants by mutating KIR2DL5A*001 and found that all of them were recognized by our mAbs (Figure 1D and Supplemental Figure 2B), including F8B30 with a much lower half maximal effective concentration (EC_50_; ranging from 8.6 to 43.6 nM) than that of UP-R1 (ranging from 391.1 to 875.9 nM) (Table 1). Collectively, our results showed that our new mAbs against the D0 domain of KIR2DL5 recognized different KIR2DL5 alleles efficiently.

### KIR2DL5 protein was expressed on human innate and adaptive immune cells.

**Figure 2:**
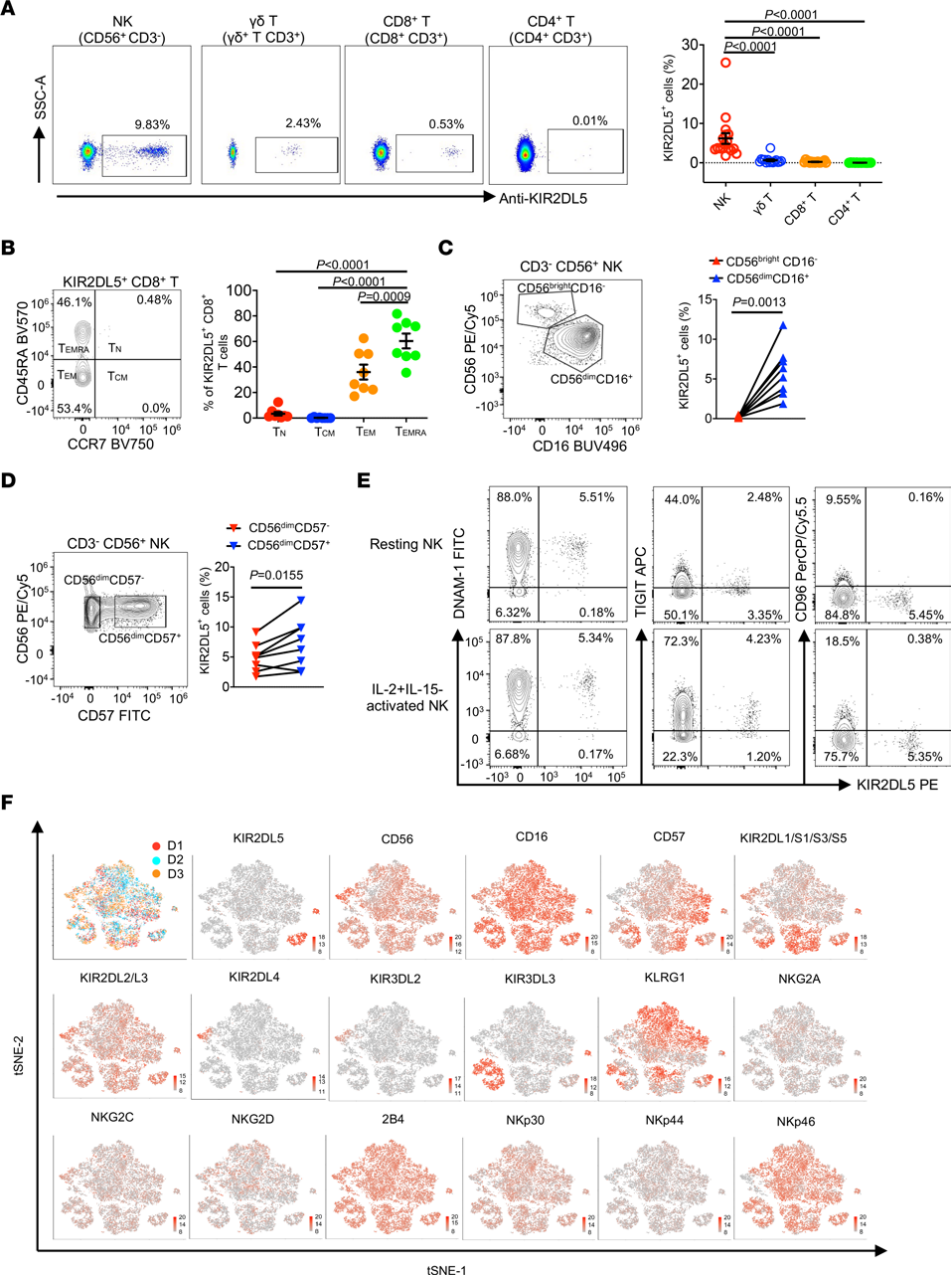
KIR2DL5 was expressed on human innate and adaptive immune cells. (**A**) KIR2DL5 expression on human PBMCs. Left: Flow cytometric analysis of KIR2DL5 expression on the indicated subsets from 1 donor. Right: The frequencies of KIR2DL5^+^ cells in the indicated subsets (*n* = 17 for NK and CD8^+^ T; *n* = 15 for CD4^+^ T and γδ T). Data are represented as mean ± SEM. (**B**) Left: The distribution of KIR2DL5^+^ CD8^+^ T cells on the indicated cell subsets based on CD45RA and CCR7 expression. Right: Summary of KIR2DL5^+^ CD8^+^ T cell distribution (*n* = 8). Data are represented as mean ± SEM. (**C**) KIR2DL5 expression on CD56^bright^CD16^–^ and CD56^dim^CD16^+^ NK subsets. The frequencies of KIR2DL5^+^ cells on the indicated NK cell subsets are shown on the right (*n* = 8). (**D**) KIR2DL5 expression on CD56^dim^CD57^–^ and CD56^dim^CD57^+^ NK subsets. The frequencies of KIR2DL5^+^ cells on the indicated NK cell subsets (*n* = 8) are shown on the right. (**E**) Flow cytometric analysis of coexpression pattern of KIR2DL5 with DNAM-1, TIGIT, and CD96 on primary resting or IL-2+IL-15–activated NK cells. (**F**) The coexpression pattern of KIR2DL5 with other receptors on NK cells from human PBMCs. The *t*-SNE plots were generated based on spectral flow cytometric data (*n* = 3). In **E**, data are representative of 3 independent experiments with 3 different donors. *P* values were determined by 1-way ANOVA (**A** and **B**) or 2-tailed paired *t* test (**C** and **D**).

Given the outperformance of our mAbs over UP-R1 for KIR2DL5 recognition, we then used F8B30 to redefine the KIR2DL5 expression pattern in human immune cells. KIR2DL5 protein was expressed on both innate (NK and γδ T cells) and adaptive (CD8^+^ T cells) immune cells from human peripheral blood (Figure 2A and Supplemental Figure 3A). Additionally, KIR2DL5^+^ CD8^+^ T cells were mainly distributed in terminally differentiated (Temra) and, to a lesser extent, effector memory cell subsets, whereas KIR2DL5 expression was very low or undetectable in naive (Tn) and central memory (Tcm) CD8^+^ T cells (Figure 2B).

In agreement with its mRNA expression pattern (Supplemental Figure 3B), we found that KIR2DL5 protein was predominantly expressed on NK cells, particularly on the CD56^dim^CD16^+^ NK subset (Figure 2C), which is more differentiated and cytolytic than the CD56^bright^CD16^–^ subset (44). CD57 defines a functionally distinct NK cell population that is highly mature and terminally differentiated (45). We found that a higher proportion of CD56^dim^CD57^+^ cells expressed KIR2DL5, as compared with the CD56^dim^CD57^−^ NK subset (Figure 2D). Stimulatory cytokines, such as IL-2, IL-12, IL-15, and IL-18, drive NK cell activation and maturation (46). TIGIT, DNAM-1, and CD96 are well-established receptors for PVR. We found that TIGIT and CD96, but not KIR2DL5, were upregulated in response to exogenous stimulation with IL-2 and IL-15 (Figure 2E). Moreover, KIR2DL5 could be coexpressed with DNAM-1 and TIGIT, whereas its expression was mutually exclusive from CD96 expression on both resting and activated NK cells (Figure 2E). Lastly, analysis of NK cell receptors by high-dimensional flow cytometry revealed that KIR2DL5 was clonally distributed in CD56^dim^CD16^+^ NK cells and was coordinately expressed with the other NK cell receptors and KIRs (Figure 2F). Altogether, these findings demonstrated that KIR2DL5 protein was predominantly expressed on NK cells with mature phenotype and cytolytic function. Furthermore, KIR2DL5 exhibited a distinct expression pattern compared with other PVR receptors, suggesting that KIR2DL5 might have a unique function.

### Allelic polymorphism of KIR2DL5 affected its interaction with PVR.

**Figure 3:**
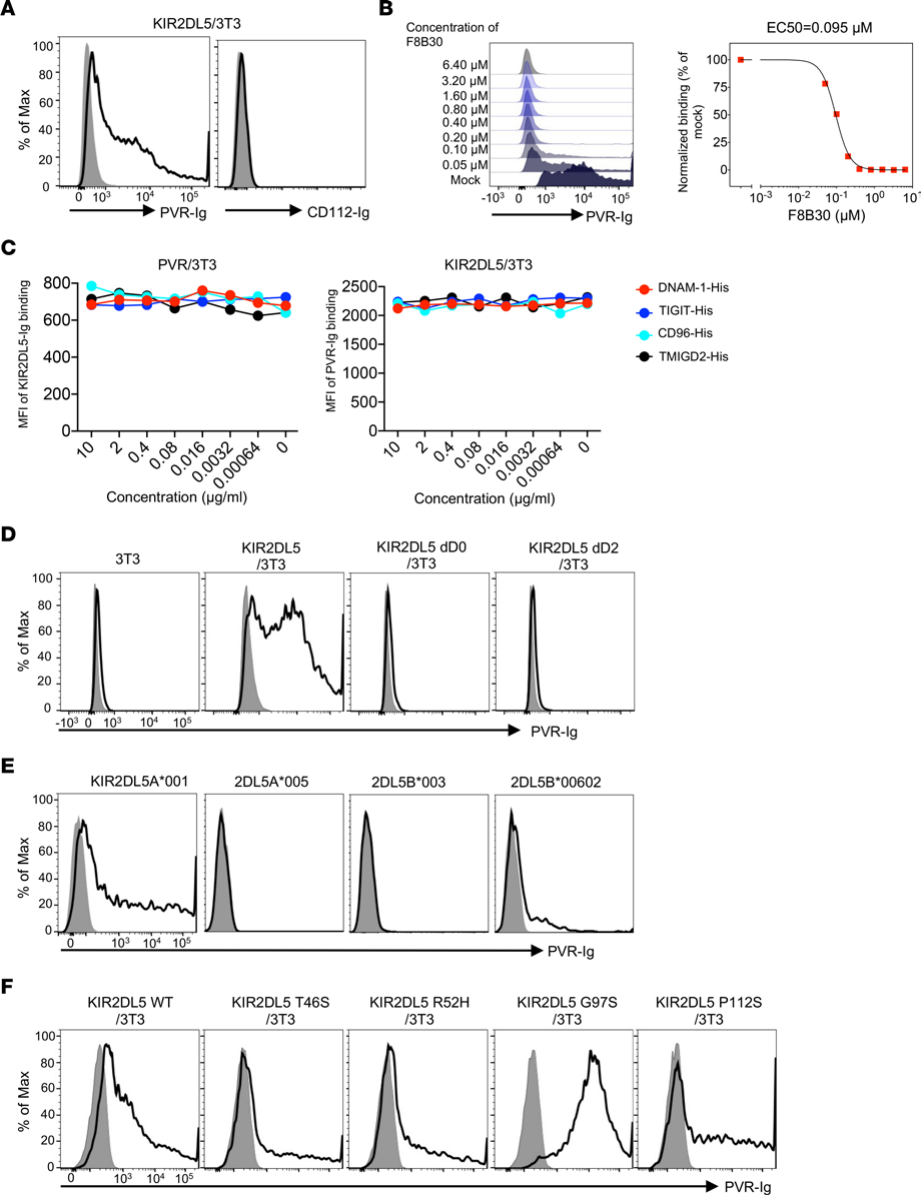
Allelic polymorphism affected PVR binding of KIR2DL5. (**A**) Flow cytometric analysis of PVR-Ig or CD112-Ig (open) or control hIg (shaded) binding to KIR2DL5/3T3. (**B**) Flow cytometric analysis of PVR-Ig binding to KIR2DL5/3T3 in the presence of increasing concentrations of F8B30. (**C**) KIR2DL5 bound to different sites of PVR from other receptors. Left: PVR/3T3 cells were preincubated with DNAM-1–His, CD96-His, TIGIT-His, or TMIGD2-His (negative control) tag protein at the indicated concentrations and then stained by KIR2DL5-Ig fusion protein. Right: PVR-Ig protein was preincubated with indicated His-tagged protein and then stained KIR2DL5/3T3 cells. (**D**) Flow cytometric analysis of PVR-Ig (open) or control hIg (shaded) binding on 3T3 cells expressing WT, D0-deleted, or D2-deleted KIR2DL5. Parental 3T3 cells were used as a negative control. (**E**) Flow cytometric analysis of PVR-Ig or control hIg (shaded) binding on 3T3 cells expressing different KIR2DL5 alleles. (**F**) Flow cytometric analysis of PVR-Ig (open) or control hIg (shaded) binding on 3T3 cells expressing WT KIR2DL5 and indicated D0 domain variants. In **A**–**F**, data are representative of 2 independent experiments.

To further characterize KIR2DL5 as a new receptor for PVR, we performed a cell-based binding assay by incubating PVR-Ig fusion protein with KIR2DL5- or KIR2DL4-expressing 3T3 cells. We observed that PVR bound to KIR2DL5 in a dose-dependent manner but not to its closest homolog, KIR2DL4 (Supplemental Figure 4A). Conversely, KIR2DL5 was selectively bound by PVR, but not by CD112 (also known as nectin-2), another ligand for TIGIT and DNAM-1 in the nectin/nectin-like family (Figure 3A). Furthermore, our anti-KIR2DL5 mAb F8B30 was able to effectively block KIR2DL5-PVR interaction (EC_50_ = 0.095 μM) (Figure 3B). The specificity of KIR2DL5 binding to PVR was also evidenced by an intercellular interaction assay, in which 3T3 cells expressing PVR interacted with 3T3 cells expressing KIR2DL5, but not with cells expressing KIR3DL3 (Supplemental Figure 4B), a newly identified inhibitory receptor of HHLA2 (47–49). As expected, KIR3DL3/3T3 cells interacted with HHLA2/3T3 but not with PVR/3T3 cells (Supplemental Figure 4B). Finally, the interaction between KIR2DL5/3T3 and PVR/3T3 was blocked by some of our anti-KIR2DL5 mAbs (Supplemental Figure 4C).

A previous study demonstrated that PVR receptors DNAM-1, TIGIT, and CD96 share a common binding site on PVR (33). We then sought to compare the binding of KIR2DL5 with these known receptors. In competition studies, we observed that DNAM-1, TIGIT, and CD96 receptors did not block the interaction of PVR with KIR2DL5 (Figure 3C), indicating that KIR2DL5 bound to PVR through a nonidentical site compared with other PVR receptors.

Classical KIR2DL1–3 recognizes HLA-C allotypes through Ig-like D1–D2 domains (18). To determine the binding pattern of KIR2DL5 to PVR, we incubated PVR-Ig with 3T3 cells expressing truncated KIR2DL5 protein. The deletion of either D0 or D2 alone completely abrogated its binding to PVR (Figure 3D), suggesting that both D0 and D2 domains contribute to the KIR2DL5-PVR interaction. We then asked whether allelic polymorphism affected PVR binding to KIR2DL5. Compared with the solid binding for 2DL5A*001, PVR weakly bound to cell surface–expressed 2DL5B*00602 but not 2DL5A*005 or 2DL5B*003 (Figure 3E). Interestingly, a serine substitution for glycine-97 in the D0 domain (G97S) significantly enhanced the PVR-Ig binding to KIR2DL5, whereas the other D0 variants showed a minor effect on PVR-KIR2DL5 binding (Figure 3F). Together, these results demonstrated that allelic polymorphism of KIR2DL5 influenced its capability of binding to PVR and that KIR2DL5 bound to PVR without competition with other known PVR receptors.

### KIR2DL5 inhibited NK cell function and mediated PVR^+^ tumor immune resistance.

**Figure 4:**
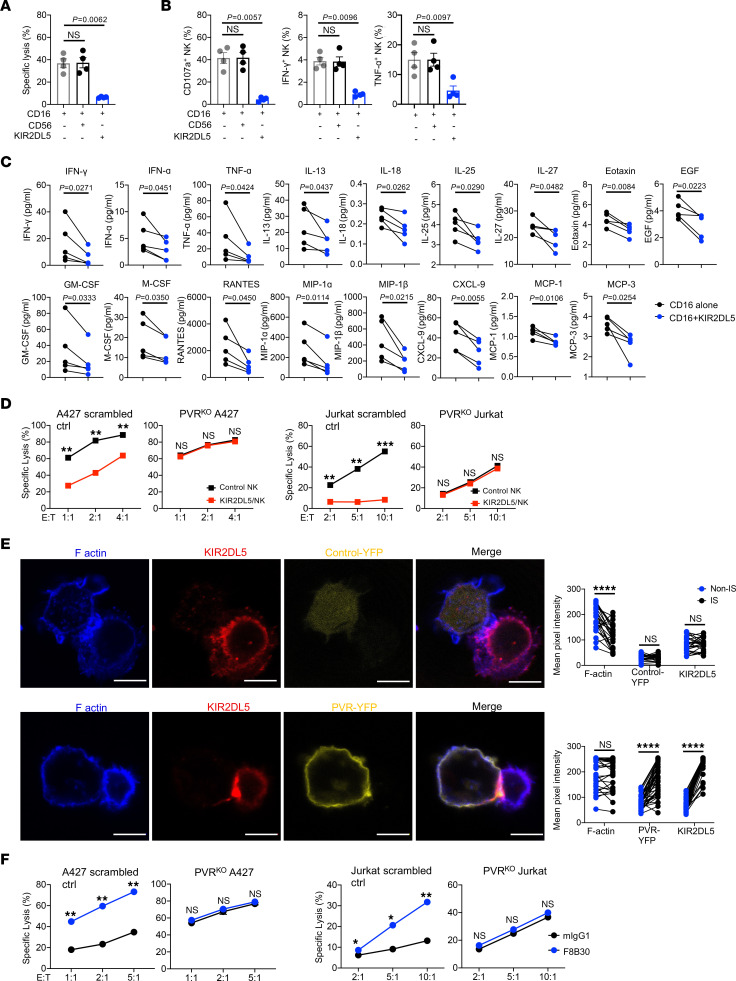
KIR2DL5 inhibited NK cell function and mediated PVR^+^ tumor immune resistance. (**A**–**C**) Redirected cytotoxicity of KIR2DL5^+^ primary NK cells against P815. (**A**) The lysis of P815 cells (*n* = 4). (**B**) The degranulation (CD107a) and cytokine production (IFN-γ and TNF-γ) of KIR2DL5^+^ primary NK cells (*n* = 4). CD56 served as negative control. (**C**) Cytokine production in the coculture supernatant of KIR2DL5^+^ primary NK cells with the indicated antibody-coated P815 (*n* = 5). Data are represented as mean ± SEM. (**D**) Lysis of scrambled control or PVR^KO^ A427 or Jurkat cells by KIR2DL5-transduced primary NK cells (KIR2DL5/NK) or KIR2DL5^–^ control NK cells (Control NK) at indicated E/T ratios. (**E**) PVR-KIR2DL5–mediated inhibitory synapse formation. Left: Representative imaging of cell conjugates acquired upon sorted KIR2DL5^+^ primary NK contact with control-YFP/Raji (top) or PVR-YFP/Raji (bottom), followed by staining with anti-KIR2DL5 mAbs and phalloidin. Scale bars: 10 μm. Right: Intensity quantification of F-actin, YFP, and KIR2DL5 at the immunological synapses (IS) and the cell surface away from synapses (Non-IS) from KIR2DL5^+^ NK cell–Control Raji (*n* = 25) and KIR2DL5^+^ NK-PVR/Raji (*n* = 35) conjugates. (**F**) Lysis of scrambled control or PVR^KO^ A427 (top) or Jurkat (bottom) cells by sorted KIR2DL5^+^ primary NK cells in the presence of F8B30 or mIgG1 at indicated E/T ratios. In **D** and **F**, data are mean for duplicate measurements and representative of 3 independent experiments with 3 different donors. *P < 0.05, **P < 0.01, ***P < 0.001, ****P < 0.0001, by 1-way ANOVA (**A** and **B**), 2-tailed paired Student’s *t* test (**C** and **E**), or multiple unpaired *t* test (**D** and **F**).

To validate whether KIR2DL5 could directly inhibit primary NK cell functions, we sorted out KIR2DL5^+^ NK cells from human PBMCs and confirmed stable KIR2DL5 expression after activation and expansion (Supplemental Figure 5A). High expression of other immune inhibitory receptors, including KIR2DL1–3, TIGIT, CD96, and TIM3, as well as the immune stimulatory receptor NKG2D, was also detected on those expanded KIR2DL5^+^ NK cells (Supplemental Figure 5B). We then employed an NK cell–based, CD16-induced redirected cytotoxicity assay and found that the co-engagement of CD16 with KIR2DL5, but not with CD56, significantly inhibited target cell P815 killing and NK cell degranulation (CD107a) as well as IFN-γ and TNF-α production (Figure 4, A and B). By performing a 65-plex human cytokine/chemokine array experiment, we observed that KIR2DL5 markedly decreased the production of a broad spectrum of cytokines/chemokines, including IL-13, IL-18, IL-25, IL-27, eotaxin, EGF, GM-CSF, M-CSF, RANTES, MIP-1α, MIP-1β, CXCL-9, and others (Figure 4C).

We next sought to examine the effect of the KIR2DL5-PVR engagement on NK-mediated tumor cell lysis. We transduced primary NK cells with KIR2DL5 (Supplemental Figure 5C) and cocultured with human lung cancer A427 and leukemic Jurkat tumor cells that expressed endogenous PVR. A427 and Jurkat cells displayed a distinct expression profile of ligands for NK cell receptors (Supplemental Figure 5, D and E) and were susceptible to NK cell killing. While the presence of KIR2DL5 dramatically suppressed NK cytolytic activity against PVR^+^ tumor cells (scrambled control), this effect was eliminated upon the deletion of PVR in tumor cells by CRISPR/Cas9 (PVR^KO^) (Figure 4D and Supplemental Figure 5, F and G). A similar observation was obtained with another leukemic tumor cell line, K562 (Supplemental Figure 5, H and I).

Formation of the NK lytic immunological synapse at the interface with the target cell facilitates NK cytotoxicity (50), whereas inhibitory receptors such as KIRs can establish the inhibitory synapse by engaging with their ligands and therefore interfere with the function of lytic synapse (51). To investigate whether KIR2DL5-PVR interaction mediated inhibitory synapse formation, we incubated primary KIR2DL5^+^ NK cells with Raji cells expressing PVR-YFP (PVR/Raji) or control-YFP (Control Raji) fusion protein (Supplemental Figure 5J). In the absence of PVR on the target cells, we observed that KIR2DL5 distributed evenly on the NK cell surface while F-actin accumulated at the interface, indicating the formation of a lytic synapse (Figure 4E, top). By contrast, in the presence of PVR on the target cells, KIR2DL5 clustering with PVR, but no F-actin polarization, was observed at the NK-Raji interface (Figure 4E, bottom), indicating the impairment of actin reorganization and the formation of an inhibitory synapse.

We next examined whether direct blockade of KIR2DL5 could enhance NK cell functions against PVR^+^ human tumors. Anti-KIR2DL5 mAb F8B30, which was able to effectively block KIR2DL5-PVR interaction, significantly enhanced the tumor lysis by KIR2DL5^+^ primary NK cells (Figure 4F, scrambled control). The effect of F8B30 was also dependent on PVR, as this mAb lost the enhanced effect on NK functions in the absence of PVR (Figure 4F, PVR^KO^). Taken together, these results demonstrated that KIR2DL5 inhibited NK cell function and facilitated tumor immune evasion through the engagement with PVR on tumor cells.

### KIR2DL5-induced inhibitory signaling in NK cells.

**Figure 5:**
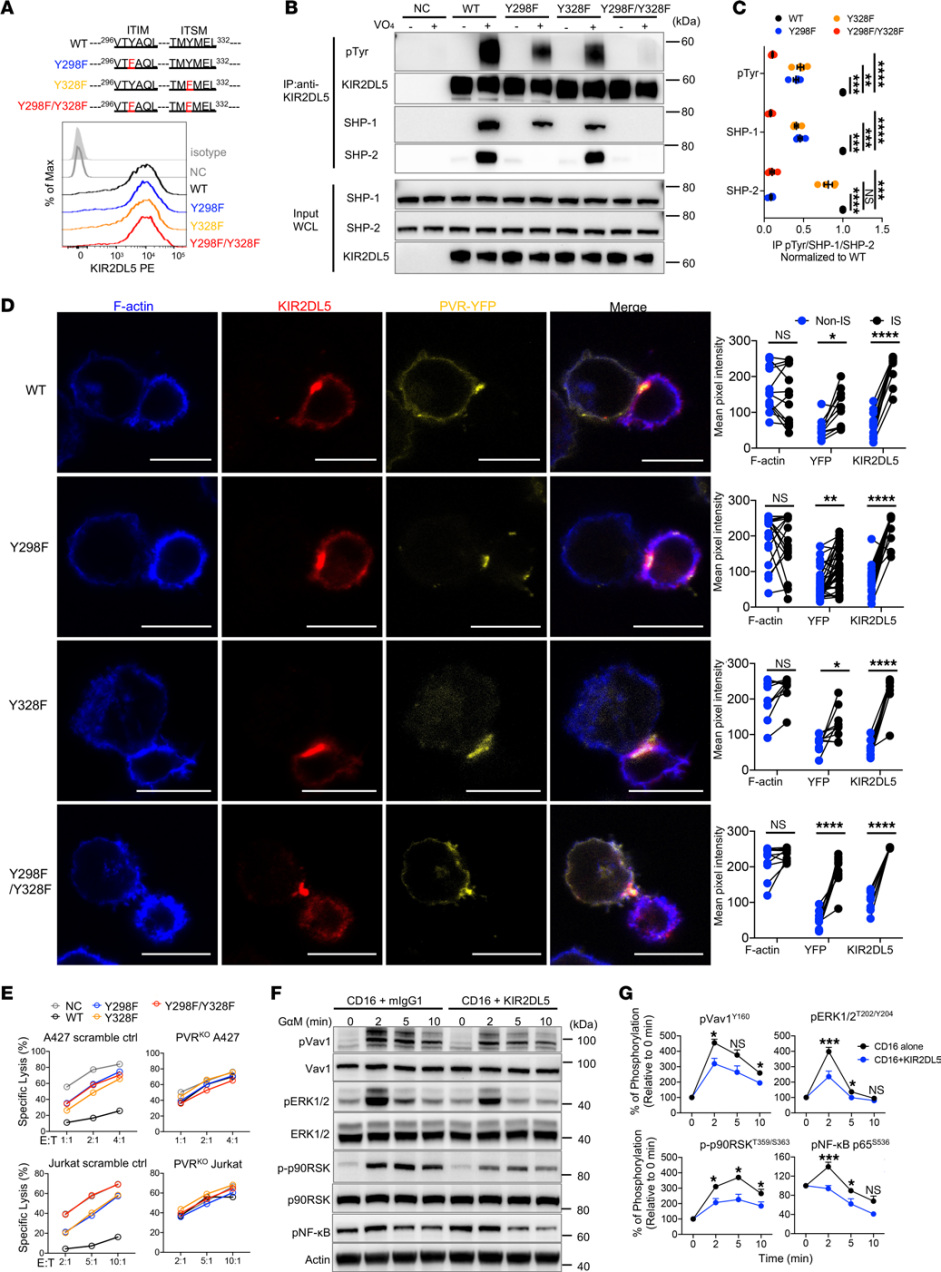
KIR2DL5 ITIM and ITSM mediated NK cell inhibition and suppressed downstream signaling. (**A**) Tyrosine (Y) in ITIM and ITSM of KIR2DL5 was mutated to phenylalanine (F). The KIR2DL5^–^ primary NK cells were transduced with WT KIR2DL5 or the indicated mutants, and then examined for protein expression with F8B30 (open) or mIgG1 (shaded). NC, negative control. Data are representative of 2 independent experiments. (**B** and **C**) Transduced primary NK cells were treated with (+) or without (–) pervanadate (VO_4_) for 5 minutes. Cell lysates were immunoprecipitated with anti-KIR2DL5 antibodies. Phospho-tyrosine (4G10), SHP-1, SHP-2, and total KIR2DL5 were detected by immunoblots (**B**). Quantification of p-Tyr, SHP-1, and SHP-2 association with WT or mutant KIR2DL5 in VO_4_-treated NK cells (**C**). WCL, whole-cell lysates. (**D**) Representative imaging of cell conjugates acquired upon the indicated transduced primary NK and PVR/Raji cell contact followed by staining with anti-KIR2DL5 mAb and DAPI. Scale bars: 10 μm. (**E**) Lysis of scramble control or PVR^KO^ A427 (top) and Jurkat (bottom) by WT or mutant KIR2DL5–transduced primary NK cells at the indicated E/T ratios. Data are mean for duplicate measurements and representative of 3 independent experiments with 3 different donors. (**F** and **G**) Expression and phosphorylation of Vav1, ERK1/2, p90RSK, and NF-κB in sorted KIR2DL5^+^ primary NK cells after cross-linking with indicated mAbs at indicated time points (**F**). Quantification of immunoblotting (**G**). GαM, goat anti–mouse IgG antibody. Data are mean ± SEM from 2 independent experiments. In **A**, **B**, and **F**, data are representative of 3 independent experiments. *P < 0.05, **P < 0.01, ***P < 0.001, ****P < 0.0001, by 1-way ANOVA (**C**), paired (**D**) or unpaired Student’s *t* test (**G**).

Since we demonstrated the inhibitory function of the KIR2DL5/PVR pathway, we next dissected the KIR2DL5-mediated signaling within primary NK cells. The cytoplasmic tail of KIR2DL5 possesses a classical ITIM and an ITSM (Supplemental Figure 1A). Substantial evidence indicates that phosphorylated ITIMs and ITSMs mediate inhibition by recruiting SHP-1 and/or SHP-2 (52). To gain insights into the role of these motifs in transducing KIR2DL5-inhibitory signaling, we mutated the tyrosine residues into phenylalanine in the ITIM (Y298F) or ITSM (Y328F) or both (Y298F/Y328F) (Figure 5A). WT KIR2DL5 and mutated KIR2DL5 were transduced into KIR2DL5^–^ primary NK cells, and their expression levels were similar after cell sorting (Figure 5A). To determine tyrosine phosphorylation and the contribution of ITIM and ITSM to KIR2DL5 association with SHP-1 and SHP-2 after mutation, we performed coimmunoprecipitation assays with primary NK cells expressing WT or mutated KIR2DL5 proteins. Upon treatment with the tyrosine phosphatase inhibitor pervanadate (53), WT KIR2DL5 exhibited tyrosine phosphorylation, whereas these mutants displayed diminished or even abrogated tyrosine phosphorylation (Figure 5, B and C). In line with previous studies (41, 54), we validated that both SHP-1 and SHP-2 were recruited by WT KIR2DL5 in primary NK cells (Figure 5B). Intriguingly, we further found that the KIR2DL5 association with SHP-1 was impaired by the tyrosine mutation in either ITIM or ITSM (Figure 5, B and C). SHP-2 recruitment by KIR2DL5 was completely abolished by ITIM tyrosine mutation, whereas it was not altered by ITSM tyrosine mutation (Figure 5, B and C). Furthermore, we found that these mutations did not affect the clustering of KIR2DL5 with PVR at the interface of immunological synapses (Figure 5D). However, PVR-KIR2DL5 interaction–mediated inhibition of NK cytotoxicity was impaired when ITIM or ITSM alone, or both, were mutated (Figure 5E).

We then investigated KIR2DL5-mediated downstream signaling. For this purpose, we conducted a receptor cross-linking assay to initiate KIR2DL5 signaling in CD16-stimulated primary NK cells and then subjected them to a human phospho-kinase array. Compared with CD16 alone, coengagement of KIR2DL5 with CD16 displayed a reduced phosphorylation level of multiple kinases, including ERK1/2 and p90RSK (Supplemental Figure 6, A and B). Further immunoblot analysis showed decreased activation of Vav1, ERK1/2, p90RSK, and the downstream transcription factor NF-κB upon KIR2DL5 signaling initiation (Figure 5, F and G).

Collectively, these results suggested that both ITIM and ITSM of KIR2DL5 underwent tyrosine phosphorylation after engagement, which was not necessary for the clustering of KIR2DL5 at the interface of immunological synapses but was essential for KIR2DL5-mediated NK suppression by recruiting SHP-1 and/or SHP-2; and that ITIM/SHP-1/SHP-2 and ITSM/SHP-1 subsequently downregulated the Vav1/ERK1/2/p90RSK and downstream NF-κB signaling pathway.

### KIR2DL5^+^ immune cells infiltrated in various PVR^+^ human cancers.

**Figure 6:**
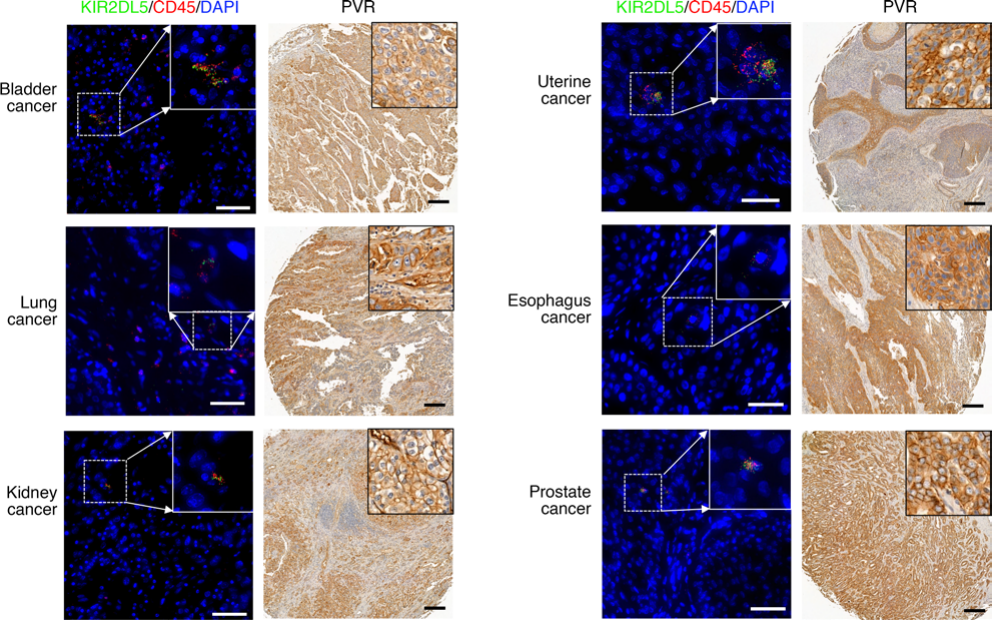
KIR2DL5^+^ immune cells infiltrated in various PVR^+^ human cancers. Representative images of the coexpression of KIR2DL5 and CD45 mRNA detected by RNAScope (left) and PVR protein expression detected by IHC (right) in the indicated cancer types. The gates in top right of the RNAScope images showed coexpression of KIR2DL5 (green) and CD45 (red) mRNA in indicated human cancers. Scale bars: 50 μm for RNAScope images and 200 μm for IHC images.

To further understand the KIR2DL5/PVR pathway within the human tumor microenvironment, we analyzed data sets from the Gene Expression Omnibus database and BloodSpot databases. We found that KIR2DL5A mRNA was upregulated in several human solid tumors and hematopoietic malignancies by comparison with respective normal tissues (Supplemental Figure 7, A and B), whereas the expression of other receptors, TIGIT, CD96, and DNAM-1, showed inconsistent change in these tumors when compared with respective normal tissues (Supplemental Figure 7A). To further explore the KIR2DL5/PVR pathway in various human cancers, we first tried immunohistochemistry (IHC) staining for KIR2DL5, but none of the antibodies worked. We then used RNAScope in situ hybridization (36) to examine KIR2DL5 mRNA expression on human tumor tissue microarrays (TMAs) with KIR2DL5-specific probes. The probe set for KIR2DL5A specifically stained KIR2DL5^+^ NK cells, but not KIR2DL5^–^ PBMCs (Supplemental Figure 7C). KIR2DL5^+^ CD45^+^ tumor-infiltrating immune cells were observed in a broad spectrum of human cancers (Figure 6 and Table 2). Next, we looked at PVR protein expression in these tumors. IHC staining showed that PVR protein was widely expressed in those cancers (Figure 6 and Table 2). These results demonstrated the presence of the immunosuppressive KIR2DL5/PVR pathway within the TME of various human cancers of bladder, kidney, breast, lung, liver, cerebrum, prostate, colon, esophagus, pancreas, uterus, and stomach, which tumors may exploit as an immune evasion mechanism.

### Blockade of KIR2DL5-PVR augmented NK cell–based antitumor immunity in vivo.

**Figure 7:**
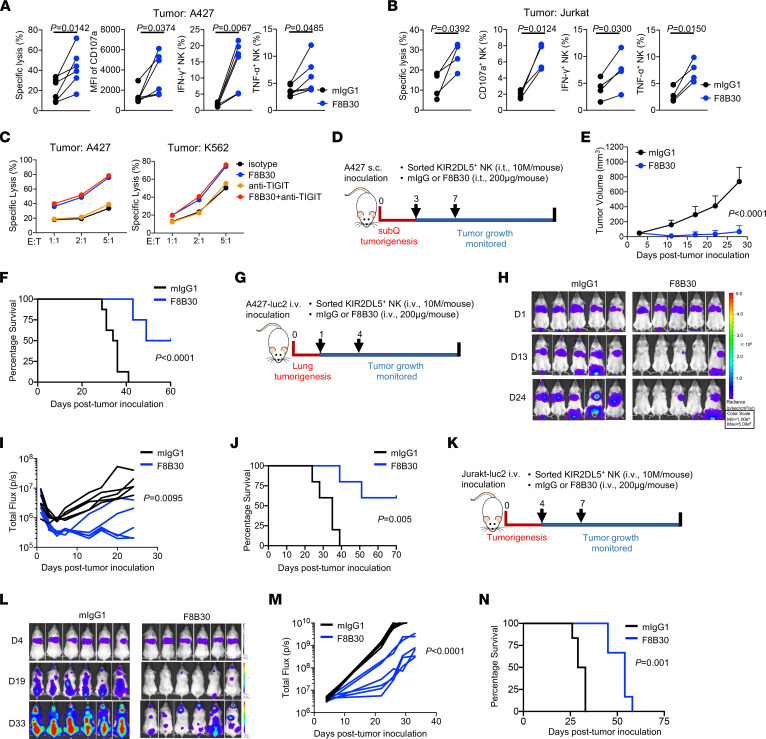
KIR2DL5 blockade promoted NK-based antitumor immunity. (**A** and **B**) KIR2DL5 blockade enhanced NK cell function in vitro. Sorted KIR2DL5^+^ primary NK cells preincubated with mIgG1 or F8B30 were cocultured with A427 (**A**) or Jurkat (**B**) tumor cells at E/T of 2:1 and 5:1, respectively. Tumor cell lysis and the degranulation (CD107a) and cytokine production (IFN-γ and TNF-γ) of NK cells from different donors (*n* = 6 for A427, *n* = 4 for Jurkat) are shown. (**C**) Lysis of A427 and K562 cells by sorted primary KIR2DL5^+^ NK cells in the presence of indicated mAbs at indicated E/T ratios. Data are mean for duplicate measurements and representative of 3 independent experiments with 3 different donors. (**D**–**F**) Subcutaneous A427 tumor mode with sorted primary KIR2DL5^+^ NK cells. (**D**) Schematic of experimental design. (**E**) Growth of A427 tumors. *n* = 8 tumors per group. (**F**) Kaplan-Meier survival curves of mice. (**G**–**J**) A427 lung metastasis model with sorted primary KIR2DL5^+^ NK cells. (**G**) Schematic of experimental design. (**H** and **I**) Tumor growth was monitored by means of bioluminescence imaging. *n* = 5 mice per group. (**J**) Kaplan-Meier survival curves of mice. (**K**–**N**) Jurkat metastasis model with sorted primary KIR2DL5^+^ NK cells. (**K**) Schematic of experimental design. (**L** and **M**) Tumor growth was monitored by means of bioluminescence imaging. *n* = 6 mice per group. (**N**) Kaplan-Meier survival curves of mice. In **D**, **G**, and **K**, data are representative of 2 independent experiments. *P* values were determined by 2-tailed paired Student’s *t* test (**A** and **B**), 2-way ANOVA (**E**, **I**, and **M**), or log-rank test (**F**, **J**, and **N**). i.t., intratumorally.

Since we demonstrated the immune inhibitory function of the KIR2DL5/PVR pathway and its presence within the TME of various human cancers, we wanted to develop a new cancer immunotherapy by targeting this pathway. Upon incubation with anti-KIR2DL5 blocking mAb F8B30, KIR2DL5^+^ NK cells manifested more potent cytotoxicity, degranulation (CD107a), and functional cytokine (IFN-γ and TNF-α) production after coculturing with PVR^+^ A427 (Figure 7A) or Jurkat tumor cells (Figure 7B). TIGIT expression was low in resting NK cells but elevated upon activation with IL-2 and IL-15 (Supplemental Figure 2E). Blockade of TIGIT on activated NK cells could promote NK degranulation (Supplemental Figure 8A), confirming its inhibitory role in regulating NK cell functions. Despite the high expression of TIGIT on KIR2DL5^+^ NK cells (Supplemental Figure 5B), our results demonstrated no change in NK cytotoxicity when TIGIT alone was blocked. Enhanced tumor lysis and NK degranulation were only observed when KIR2DL5 was blocked, either alone or with TIGIT blockade (Figure 7C and Supplemental Figure 8B), suggesting that KIR2DL5 has a dominant role over TIGIT in inhibiting KIR2DL5^+^TIGIT^+^ NK cell cytotoxicity.

We next sought to inverstigate whether the enhancement of NK cell function by KIR2DL5 blockade could be recapitulated in vivo. Since mice do not express a KIR2DL5 homolog, we decided to use humanized nonobese diabetic (NOD). Cg-Prkdc^scid^Il2rg^tm1Wjl^/SzJ (NSG) mouse models. We initially used a subcutaneous tumor model in which NSG mice were engrafted with A427 cells and then reconstituted with KIR2DL5^+^ primary NK cells intratumorally, followed by F8B30 or isotype control treatment (Figure 7D). Compared with mIgG1 treatment, blockade of KIR2DL5 significantly inhibited tumor growth, as shown by significantly lower tumor volume (Figure 7E) and improved overall mouse survival (Figure 7F). Similar results were obtained using NSG–hIL-15 mice, which express human IL-15 and better support human NK cell survival after cell transfer (Supplemental Figure 8, C–E).

We next tested the antitumor efficacy of F8B30 in a more physiologically relevant lung tumor model. NSG mice were inoculated i.v. with luciferase^+^ A427 tumor cells (A427-luc2) and treated with KIR2DL5^+^ primary NK cells and F8B30 or mIgG1 (Figure 7G). Tumor growth in the lungs was monitored by bioluminescence. Compared with mIgG1-treated mice, F8B30-treated mice showed significantly slower tumor growth (Figure 7, H and I). Whereas all mIgG1-treated mice reached an endpoint within 40 days, 2 of 5 F8B30-treated mice were tumor free beyond 70 days upon tumor inoculation (Figure 7J). In line with those results, in the Jurkat-luc2 tumor model, F8B30 significantly reduced tumor dissemination and prolonged overall mouse survival after tumor inoculation and adoptive KIR2DL5^+^ NK cell transfer (Figure 7, K–N). Taken together, these results demonstrated that blockade of KIR2DL5-PVR reinvigorated NK cell function and enhanced human NK cell–based antitumor immunity in vitro and in vivo.

## Discussion

The human KIRs are critical regulators of NK cell function and are important for immunological tolerance and tumor surveillance (55). KIR2DL5 is the most recently identified KIR molecule and has been reported to inhibit NK cell function against the P815 cell line in a redirected cytotoxicity assay (41). However, the mechanisms of action of KIR2DL5 remain unexplored. A nectin/nectin-like family protein, PVR, was recently identified as a binding partner for KIR2DL5 (23, 24), but the physiological role of the KIR2DL5/PVR pathway in tumor immunity and the therapeutic potential targeting this newly identified pathway have yet to be fully elucidated. In the present study, we demonstrated that KIR2DL5 suppressed primary NK cell cytotoxicity against multiple solid and hematopoietic tumor cells in a PVR-dependent manner. We further revealed KIR2DL5-induced inhibitory signaling in primary NK cells. Blockade of KIR2DL5 with our new blocking mAb significantly enhanced NK-mediated antitumor immunity both in vitro and in vivo, demonstrating blockade of the KIR2DL5/PVR pathway as an immunotherapy for treating human cancers.

The identification of KIR2DL5 as an inhibitory receptor of PVR adds KIR2DL5 into a complex regulatory network composed of the other 2 inhibitory receptors, TIGIT and CD96, and 1 activating receptor, DNAM-1, for PVR. Unlike TIGIT and CD96, which share a common binding site with DNAM-1 on PVR (33), we found that KIR2DL5 bound to a non-identical site on PVR and did not compete with those 3 receptors for PVR binding, suggesting a distinct mechanism by which KIR2DL5 exerts an inhibitory effect through engagement with PVR. We demonstrated that KIR2DL5 mediated PVR^+^ tumor immune resistance to NK cell killing. Furthermore, KIR2DL5-mediated inhibition on NK cytotoxicity was abolished upon depletion of PVR on tumor cells, corroborating that KIR2DL5 acts via PVR. These findings support PVR as a primary ligand for KIR2DL5 to induce NK cell suppression and tumor immune evasion.

Allelic polymorphism significantly influences cell surface expression, antibody recognition, and ligand avidity of KIRs (56, 57). Distinct from UP-R1, which required both D0 and D2 domain for KIR2DL5 recognition, our anti-KIR2DL5 mAb F8B30 bound to KIR2DL5 through the D0 domain, suggesting that they recognize different epitopes on KIR2DL5. Indeed, we observed that, besides 2DL5A*001 and D0 variants, F8B30 could also detect surface-expressed 2DL5A*005, the second most common 2DL5A allele in the human population, while UP-R1 failed to do so. Furthermore, we also demonstrated that PVR displayed a different binding capacity to different KIR2DL5 alleles. In comparison with 2DL5A*001, 2DL5B*00602 was moderately bound by PVR, while surface-expressed 2D5A*005 and 2DL5B*003 were not bound by PVR. It remains to be elucidated whether the polymorphism impacts the inhibitory function of KIR2DL5 in tumor immune response.

Crosstalk between NK cells and dendritic cells (DCs) via cytokines or direct cell-contact stimuli results in activation and cytokine production by both cell types, contributing to the coordination of innate and adaptive immune responses (58, 59). Here our results demonstrated that KIR2DL5 could significantly decrease the production of a broad spectrum of cytokines and chemokines by NK cells, such as IFN-γ, TNF-α, and GM-CSF, which might subsequently impair NK cell–induced DC maturation and activation. PVR is highly expressed not only by tumor cells but also by some immune cell subsets, including DCs. TIGIT could induce PVR phosphorylation and signaling in DCs, resulting in increased IL-10 and decreased IL-12 production by DCs (33). DC-released IL-12 could induce IFN-γ production and potentiate the cytotoxicity of NK cells (60). Further investigation will be needed to assess whether direct KIR2DL5 engagement with PVR on DCs could also affect NK-DC interaction and immune homeostasis by initiating DC-intrinsic PVR signal, which, in turn, modulates NK cell activation and cytotoxicity.

ITIM and ITSM sequences found in many inhibitory receptors are critical in transducing negative signaling through recruiting phosphatases, such as SHP-1 or SHP-2, upon tyrosine phosphorylation (52, 61). The results of our tyrosine mutation study showed that both ITIM and ITSM were essential for KIR2DL5-mediated NK cell inhibition. KIR2DL5 recruited both SHP-1 and SHP-2 in primary human NK cells. Notably, we demonstrated that both phosphorylated ITIM and ITSM contributed to KIR2DL5 association with SHP-1. Intriguingly, KIR2DL5 association with SHP-2 completely relied on phosphorylated ITIM, but not ITSM. ITIM/SHP-1/SHP-2 and ITSM/SHP-1 inhibited the Vav1/ERK1/2/p90RSK and downstream NF-κB signaling pathway. These findings revealed the molecular basis for KIR2DL5-mediated suppression of NK cells.

Preclinical studies have demonstrated that the TIGIT/PVR axis is an attractive cancer immunotherapy target owing to its roles in modulating CD8^+^ T cell and NK cell responses (13, 33, 62). However, TIGIT blockade monotherapy shows minimal effects on controlling tumor growth. Whereas dual blockade of TIGIT and PD-1/PD-L1 shows promising results in some experimental tumor models (63–65) and in multiple trials (35–39), combination of the anti-TIGIT antibody tiragolumab and the PD-L1 inhibitor atezolizumab failed to improve progression-free survival in a phase III extensive-stage small cell lung cancer trial (ClinicalTrials.gov NCT04256421). Our results of the noncompetitive binding of KIR2DL5 and TIGIT to PVR suggested that both receptors can function simultaneously and independently and that blockade of the TIGIT/PVR axis would still leave the KIR2DL5/PVR pathway intact. In the context of our study, TIGIT blockade had a minimal effect on NK cell cytotoxicity, whereas KIR2DL5 blockade markedly restored the cytolytic activity of NK cells. Thus, the existence of KIR2DL5-mediated inhibition of NK cells in the TME represents a substantial obstacle to the success of the blockade of TIGIT. Indeed, we observed KIR2DL5^+^ immune cells infiltrated in various human cancers that highly expressed PVR. Significantly, blockade of KIR2DL5 effectively inhibited tumor growth and improved mouse survival across multiple humanized mouse models. Subsequent humanization of these anti-KIR2DL5 mAbs and testing in clinical settings will provide further evidence to support this therapeutic approach in cancer patients. In summary, our findings unraveled the cellular and molecular mechanisms underlying the inhibitory function of the KIR2DL5/PVR pathway, supporting that blockade of the immunosuppressive KIR2DL5/PVR axis alone or in combination with other therapies is a new therapeutic strategy. 

## Methods

### Mice.

BALB/c mice were purchased from Charles River Laboratory. NOD.Cg-Prkdc^SCID^Il2rg^tm1Wjl^/SzJ (NSG) and NSG-IL-15 mice were purchased from The Jackson Laboratory. Mice were used between 6 and 8 weeks of age. All mice were bred and maintained in a specific pathogen–free facility with a 12-hour light/12-hour dark cycle at Albert Einstein College of Medicine (Bronx, New York, USA).

### Cell lines.

Human cell lines used in this study include Phoenix-ampho, retrovirus producer line (ATCC, CRL-3213); HEK293T, lentivirus producer line (a gift from Wenjun Guo, Department of Cell Biology, Albert Einstein College of Medicine); K562, human chronic myelogenous leukemia (ATCC, CCL-243); Jurkat, a human T lymphoblastic leukemia cell line (ATCC, TIB-152); Raji, human B cell lymphoma (ATCC, CCL-86); and A427, human lung adenocarcinoma (a gift from Haiying Cheng, Department of Cell Biology, Albert Einstein College of Medicine). These cell lines were cultured in either EMEM, DMEM, or RPMI 1640 (Gibco) medium supplemented with 10% FBS, 100 U/mL penicillin, and 100 μg/mL streptomycin. Mouse cell lines used in this study were mouse fibroblast line NIH 3T3 (ATCC, CRL-1658), mouse mast cell line P815 (ATCC, TIB-64), and mouse myeloma cell line NSO (a gift from Matthew D. Scharff, Department of Cell Biology, Albert Einstein College of Medicine). Cells were cultured in DMEM supplemented with 10% FBS, 100 U/mL penicillin, and 100 μg/mL streptomycin. All cell lines were cultured at 37°C in a humidified atmosphere containing 5% CO_2_.

### Human phospho-kinase arrays.

The phosphorylation profiles of downstream kinases of the PVR/KIR2DL5 pathway were determined by use of a human phospho-kinase array (R&D Systems). Briefly, KIR2DL5^+^ primary NK cells (5 × 10^6^) were preincubated with 10 μg/mL isotype control mIgG1 or anti-KIR2DL5 mAbs (clone F8B10) in the presence of anti-CD16 (5 μg/mL) for 30 minutes on ice. After washing with medium, primary NK cells were cross-linked with 25 μg/mL goat anti–mouse IgG (minimal x-reactivity) (BioLegend) at 37°C water bath for 2 minutes. Cells were immediately transferred to ice to stop the reaction and then lysed with cell lysis buffer, followed by analysis of the relative levels of protein phosphorylation according to the manufacturer’s instructions.

### Production and purification of human fusion proteins.

KIR2DL5-Ig was generated in an inducible secreted serum-free *Drosophila* expression system as described previously (48, 66). Briefly, the coding region of the extracellular domain without signal peptide of KIR2DL5 was fused to a human IgG1 Fc tag in a pMT/BiP vector. Construct was cotransfected with a blasticidin-resistant plasmid into *Drosophila* Schneider 2 (S2) cells by the calcium phosphate transfection kit (Invitrogen). The stably transfected S2 cells were selected and expanded in Schneider’s *Drosophila* Medium (Gibco) supplemented with 10% FBS, 100 U/mL penicillin, 100 μg/mL streptomycin, and 25 μg/mL blasticidin (Gold Biotechnology). The S2 cells were induced to secrete fusion proteins in Express Five serum-free medium (Life Technologies) in the presence of 0.75 mM CuSO_4_. Proteins were purified using Protein G resin (GenScript) columns.

### Generation of stable cell lines.

Molecules expressed in NIH 3T3 and Raji cells were introduced by retrovirus transduction. Retrovirus was produced in Phoenix-ampho cells transfected with pCMV-VSV-G and MSCV-YFP containing the gene of interest using jetPRIME reagents (Polyplus Transfection).

Molecules expressed in A427, Jurkat, and K562 were introduced by lentiviral transduction. Lentivirus was produced in HEK293T cells transfected with pCMVR8.74, pCMV-VSV-G, and a lentiviral backbone vector containing the gene of interest using jetPRIME reagents (Polyplus Transfection). Virus-containing supernatant was harvested 48–72 hours after transfection and filtered through a 0.45 μm filter. Cells were spin-infected at 2000*g* for 120 minutes at 37°C in the presence of 5 μg/mL Polybrene (Merck Millipore) and 1–2 mL virus supernatant. Transduced cells were sorted using a BD FACSAria Fusion Cell Sorter (BD Biosciences).

### Fusion protein–cell–binding assays.

PVR-Ig, CD112-Ig, or hIgG (R&D Systems) was incubated with corresponding 3T3 cells on ice for 45 minutes, followed by incubation with APC- or PE-conjugated anti-human IgG Fc antibody (1:100; clone HP6017, BioLegend) on ice for 30 minutes. Cells were then acquired on an LSR II flow cytometer (BD Biosciences). In the anti-KIR2DL5 mAb blocking assay, KIR2DL5/3T3 cells were preincubated with a serial concentration of anti-KIR2DL5 mAb F8B30 or mIgG1 on ice for 30 minutes. After washing, the cells were then incubated with 20 μg/mL PVR-Ig or hIgG on ice for 45 minutes, followed by incubation with APC anti-human IgG Fc antibody on ice for 30 minutes. In the PVR receptor competition binding assay, PVR-YFP/3T3 cells were preincubated with recombinant human DNAM-1–His (R&D Systems), TIGIT-His (R&D Systems), or CD96-His (Thermo Fisher Scientific) tag proteins at indicated concentrations at room temperature (RT) for 40 minutes. KIR2DL5-Ig (20 μg/mL) protein was then incubated with PVR-YFP/3T3 cells on ice for 45 minutes, followed by PE anti-human IgG Fc (1:200; BioLegend) on ice for 30 minutes. In the reverse direction, PVR-Ig protein (20 μg/mL) was preincubated with indicated concentrations of His-tagged protein and then stained KIR2DL5/3T3 cells on ice for 45 minutes, followed by PE anti–human IgG Fc on ice for 30 minutes. Cells were then acquired on an LSR II (BD Biosciences).

### Intercellular conjugation assay.

PVR/3T3 and HHLA2/3T3 cells were prelabeled with eFluor 450 (eBioscience) while KIR2DL5/3T3 and KIR3DL3/3T3 were prelabeled with PKH26 (Sigma-Aldrich) to distinguish from each other. PVR/3T3 or HHLA2/3T3 cells (2 × 10^5^) were then incubated with KIR2DL5/3T3 or KIR3DL3/3T3 (2 × 10^5^) at 37°C for 45 minutes. In the mAb blocking assay, PVR/3T3 cells were coincubated with KIR2DL5/3T3 or KIR3DL3/3T3 cells in the presence of the indicated anti-KIR2DL5 mAbs or mIgG1 (10 μg/mL). After washing, cells were acquired on an LSR II (BD Biosciences) to analyze intercellular conjugation.

### Generation of mAbs against KIR2DL5.

Mouse anti-KIR2DL5 mAbs were generated by hybridoma techniques as described previously (48, 66). Briefly, splenocytes from KIR2DL5-Ig–immunized BALB/c mice were fused with NSO myeloma cells. Eight clones that specifically recognized KIR2DL5 were selected by high-throughput flow cytometry. Hybridoma cells were cultured in CELLine 350 Bioreactor Flask (DWK Life Sciences). Antibodies were purified from hybridoma supernatant by Protein G resin (GenScript) columns. The purity and integrity of antibodies were determined by SDS-PAGE and FACS. Clone F8B30 was conjugated with PE by SiteClick R-PE Antibody Labeling Kit (Invitrogen) for the following analysis.

### Biolayer interferometry.

The affinities of anti-KIR2DL5 mAbs were analyzed by biolayer interferometry using an Octet RED96 system (ForteBio, Pall LLC). Briefly, anti-human Fc capture biosensors (ForteBio, Pall LLC) were preloaded with KIR2DL5-Ig and then dipped into a solution containing mAb at 2-fold serial dilutions (from 200 to 1.5 μg/mL). Data were analyzed using Octet Data Analysis software 9.0 (ForteBio, Pall LLC). The global data fitting to a 1:1 binding model was used to estimate values for the *K*_on_ (association rate constant), *K*_off_ (dissociation rate constant), and *K_D_* (equilibrium dissociation constant).

### Immunophenotyping by flow cytometry.

Monoclonal antibodies (clone 26E10) against KIR3DL3 were purified in-house (48). The following fluorophore-conjugated antibodies were used (all antibodies from BioLegend unless otherwise indicated) (Supplemental Table 1): CD3 (clone UCHT1, BD Biosciences), CD4 (clone RPA-T4), CD8 (clone RPA-T8, BD Biosciences), CD16 (clone 3G8, BD Biosciences), CD19 (clone SJ25C1), CD56 (clone 5.1H11), anti–human CD57 (clone QA17A04), TCR γδ (clone B1), CCR7 (clone G043H7), CD45RA (clone HI100), CD155 (clone SKII.4), DNAM-1 (clone 11A8), TIGIT (clone A15153G), CD96 (clone NK92.39), CD107a (clone H4A3), IFN-γ (clone B27), TNF-α (clone MAb11), CD57 (clone HNK-1), KLRG1 (clone SA231A2), KIR3DL2 (clone 539304, R&D), KIR2DL1/S1/S3/S5 (clone HP-MA4), KIR2DL2/3 (clone DX27), KIR2DL4 (clone mAb 33), KIR2DL5 (clone UP-R1), NKG2D (clone 1D11), NKG2C (clone 134591, R&D), NKG2A (clone 131411, BD Biosciences), 2B4 (clone C1.7), NKp46 (clone 9E2), NKp44 (clone p44-8, BD Biosciences), NKp30 (clone p30-15, BD Biosciences).

Human PBMCs were stained with Zombie Violet Fixable Viability Kit (BioLegend) and then incubated with FcR blocking reagents (Miltenyi Biotec). For surface marker staining, cells were incubated with specific antibodies for 30–45 minutes at 4°C. For CD107a and intracellular cytokine staining, cells were incubated with anti-CD107a in the presence of 5 μg/mL brefeldin A and 2.5 μg/mL monensin (BioLegend) for 5 hours. Cells were then fixed and permeabilized using the Fixation/Permeabilization Solution Kit (BD Biosciences) according to the manufacturer’s instructions, followed by staining with intracellular antibodies for 30–45 minutes at 4°C. All samples were acquired on an LSR II (BD Biosciences) or Aurora (Cytek) and were analyzed using FlowJo software (BD Biosciences). DownSample and *t*-distributed stochastic neighbor embedding (*t*-SNE) plugins in FlowJo and ggplot2 package in R were used to generate *t*-SNE plots.

### Isolation and culture of human NK cells.

Human PBMCs were isolated from the buffy coats of healthy donors purchased from New York Blood Center, using Ficoll-Hypaque (GE Healthcare) density gradient separation. Human KIR2DL5^+^ primary NK cell were sorted by FACS and then expanded by culturing with autologous PBMCs as feeder cells (irradiated at 30 Gy, feeder cells: NK cells = 20:1) in OpTimizer (Invitrogen) supplemented with 5% human AB serum (Sigma-Aldrich), 1% l-glutamine, 100 U/mL penicillin, 100 μg/mL streptomycin, anti-CD3 OKT3 (10 ng/mL; BioLegend), recombinant human IL-2 (40 ng/mL; BioLegend), and IL-15 (10 ng/mL; BioLegend). Five or six days later, NK cells were further expanded in the same medium without anti-CD3 and feeder cells.

### Primary NK cell transduction.

KIR2DL5 wild type and variants of ITIM/ITSM expressed on the surface of KIR2DL5^–^ primary NK cells were introduced by lentiviral transduction. Lentivirus was produced in HEK293T cells cotransfected with psPAX, pMD2.G, and a lentiviral backbone pSin vector (a gift from the Alec Zhang laboratory, Department of Physiology, University of Texas Southwestern Medical Center, Dallas, Texas, USA) containing the full-length gene sequence of KIR2DL5A*001 using jetPRIME reagents (Polyplus Transfection). Virus-containing supernatant was harvested 48–72 hours after transfection and filtered through a 0.45 μm filter. Non-tissue-culture-treated plates were coated with retronectin, and virus supernatant was then incubated on the surface of plates at 2,000*g* for 120 minutes at 37°C. NK cells were subsequently spun down at 1000*g* for 10 minutes at 37°C. Transduced NK cells were sorted using a BD FACSAria Fusion Cell Sorter (BD Biosciences).

### Cytotoxicity assay.

Cytotoxicity assays were performed through a flow-based assay. Briefly, target cells were labeled with PKH26 (Sigma-Aldrich) for 2 minutes at 37°C. For mAb blocking assay, primary NK cells were preincubated with 20 μg/mL of mIgG1, anti-KIR2DL5 mAb (clone F8B30), anti-TIGIT mAbs (clone MBSA43, eBioscience), or indicated combination for 30 minutes before coculture with target cells.

In CD16-induced redirected cytotoxicity assays, anti–human CD16 mAbs (clone 3G8) were used to activate NK cells through cross-linking CD16. Briefly, P815 cells were preincubated with 0.5 μg/mL of anti–human CD16 and 2 μg/mL of mIgG1, anti-KIR2DL5 mAb (clone F8B30), or anti-CD56 (clone 5.1H11) for 15 minutes at RT. Target cells were coincubated with effector NK cells in 96-well round-bottom plates at indicated E/T ratios for 4–6 hours at 37°C. Supernatants from redirected cytotoxicity assays were collected after 24 hours of coculture for Human Cytokine 65-Plex Assay (Eve Technologies). 7-AAD was used to differentiate dead cells from live cells. The standard formula of 100 × PKH26^+^7-AAD^+^ cells/PKH26^+^ cells % was used to calculate specific lysis percentages.

### NK-Raji conjugation assay.

KIR2DL5^+^ primary NK or transduced NK cells (5 × 10^5^) with KIR2DL5 WT, Y298F, Y328F, or Y298/328F mutants were coincubated with 5 × 10^5^ PVR-YFP/Raji or YFP/Raji cells in a 50 mL tube at 37°C for 40 minutes. Cell mixtures were then loaded onto poly-l-lysine–precoated slides and fixed with 4% formaldehyde at RT for 15 minutes. After blocking with 5% normal goat serum at RT for 1 hour, cells were stained with 20 μg/mL anti-KIR2DL5 antibodies (a mixture of 8 homemade clones) at 4°C overnight and then with goat anti-mIgG (H+L) Alexa Flour 647 (Invitrogen) at RT for 2 hours. The cells were permeabilized by 0.1% Triton X-100 at RT for 15 minutes and stained with Alexa Flour Plus 405 Phalloidin (Life Technologies) for 1 hour at RT. The slides were then mounted by Gold Antifade Mountant without DAPI (Life Technologies). The mean pixel intensity of synapse and non-synapse was respectively measured and statistically analyzed. Images were acquired by Leica SP8 confocal microscope and processed by ImageJ (NIH).

### Plasmid construction and site-directed mutagenesis.

The plasmid encoding KIR2DL5 was purchased from Molecular Cytogenetics Core of Albert Einstein College of Medicine, and the fragment of KIR2DL5 was inserted into MSCV-YFP vector. The mutagenesis was carried out using New England Biolabs Q5 Site Directed Mutagenesis Kit. The mutants of KIR2DL5 were constructed using the following primers: deleted D0 forward, GGTCTATTTGGGAAACCTTCACTCTCAG; deleted D0 reverse, TGTCCAGGCCCCCTGCAG; deleted D2 forward, GGAAACTCTTCAAGTAGTTCATC; deleted D2 reverse, TGTGACCACGATCACCAG; N173D forward, GCCCAGCGTCGATGGAACATTCC; N173D reverse, ACTGCAGGGAGCCTAGGTT; N173D/G195S for 2DL5A*005 forward, CACATGCTTCAGCTCTCTCCATGAC; N173D/G195S for 2DL5A*005 reverse, TAGGTCCCTCCGTGGGTG; I6V forward, GCTCATGGTCGTCAGCATGGCGT; I6V reverse, GACATAGATCTAATCCGGCGC; I6V/T21P for 2DL5B*00602 forward, GGGGGCCTGGCCACATGAGGGTG; I6V/T21P for 2DL5B*00602 reverse, TGCAGCAAGAAGAACCCAACACAC; I6V/T21P/V116M for 2DL5B*003 forward, CCTGGTGATCATGGTCACAGGTC; I6V/T21P/V116M for 2DL5B*003 reverse, GGGTTGCTGGGTGCTGAC; T46S forward, GGACATGTGAGTCTTCTGTGTCGC; T46S reverse, TCCTCGAGGCACCACAGC; R52H forward, TGTCGCTCTCATCTTGGGTTTAC; R52H reverse, CAGAAGAGTCACATGTCC; G97S forward, CAGATGTCGGAGTTCACACCCAC; G97S reverse, TAGGTCCCTGCGTGTGCA; P112S forward, ACCCAGCAACTCCCTGGTGAT; P112S reverse, GCTGACCACTCAATGGGG; Y298F forward primer, GGAGGTGACATTTGCACAGTTGG; Y298F reverse primer, TGAGGGTCTTGATCATCAG; Y328F forward primer, TACCACCATGTTCATGGAACTTC; Y328F reverse primer, TCTGTTGGAGGTGTCTTG.

The following primers were used to construct pSin-KIR2DL5 WT vector and mutants: pSin forward, TGTCGTGAGGAATTGATCCTTCGAACTAGTATGTCGCTCATGGTCATCAG; pSin reverse primer, TGTAAGTCATTGGTCTTAAAGGTACCTGAGGTCAGATTCCAGCTGCTGGT.

The restriction enzyme sites were Bsu36I and SpeI.

### Lentiviral CRISPR/Cas9–induced deletion of PVR.

The scramble control sgRNA and PVR-targeting sgRNA were designed using GPP sgRNA Designer (67) (https://portals.broadinstitute.org/gpp/public/analysis-tools/sgrna-design). Oligonucleotides were annealed in T4 DNA-ligase buffer (New England Biolabs), cloned into lentiCRISPR version 2 (Addgene, 52961).

The sgRNA sequences were as follows: scrambled control sgRNA: 5′-GCACTACCAGAGCTAACTCA-3′; PVR targeting sgRNA no. 1: 5′-GATGTTCGGGTTGCGCGTAG-3′; PVR targeting sgRNA no. 2: 5′-TTGAGGGCACCAATATCCAG-3′.

All these constructs are not predicted to target any known sequences in the human genome. The lentiviruses were produced as described above. A427 and K562 were transduced with viral supernatant and then selected by puromycin (2 μg/mL) for 3 days. Stable knockout of PVR (PVR KO) was confirmed by flow cytometry analysis.

### Coimmunoprecipitation and immunoblotting.

NK92 cells or primary NK cells pretreated with or without 1 mM pervanadate (New England BioLabs) were lysed in Pierce immunoprecipitation lysis buffer supplemented with protease and phosphatase inhibitor cocktail (Thermo Fisher Scientific). Proteins from whole-cell lysis were further incubated with anti-KIR2DL5 antibodies and Dynabeads protein G (Thermo Fisher Scientific) for further immunoprecipitation. To analyze phosphorylation status, after receptor cross-linking, the cells were lysed in radioimmunoprecipitation lysis buffer (50 mM Tris-HCl [pH 7.5], 0.15 M NaCl, 1% NP-40, 0.5% sodium deoxycholate, and 0.1% SDS) supplemented with protease and phosphatase inhibitor cocktail. Samples were separated on SDS-PAGE gels (GenScript) and transferred onto nitrocellulose membranes (Bio-Rad) for protein detection.

The following antibodies were used: anti–phospho-tyrosine 4G10 (1:1,000; Sigma-Aldrich), anti–SHP-1 (1:500; Cell Signaling Technology [CST]), anti–SHP-2 (1:500; CST), anti-Vav1 (1:2,000; CST), anti–phospho-Vav1 Tyr160 (1:2,000; Invitrogen), anti-ERK1/2 (1:2,000; CST), anti–phospho-ERK1/2 Thr202/Tyr204 (1:1000; BioLegend), anti-p90RSK (1:1,000; CST), anti–phospho-p90RSK Thr359/Ser363 (1:1000; CST), anti–phospho–NF-κB p65 Ser536 (1:1,000; CST), anti–β-actin (1:2,000; Santa Cruz Biotechnology), HRP-conjugated goat anti-mouse (1:1,000; Jackson ImmunoResearch), rabbit anti-goat (1:1,000; Jackson ImmunoResearch), and goat anti-rabbit (1:2,000; CST) secondary antibodies and enhanced chemiluminescent substrate (ECL; Bio-Rad).

### RNAScope in ISH and imaging.

RNAScope ISH for KIR2DL5 and CD45 mRNA expression in FFPE human tumor tissue microarrays (TMAs; US Biomax) was performed with RNAScope 2.5 HD Reagent kit (Advanced Cell Diagnostics) per the manufacturer’s instructions (68). Briefly, TMA slides were deparaffinized, subjected to antigen retrieval using citrate buffer for 15 minutes at a boiling temperature, and then treated with 10 μg/mL protease at 40°C for 30 minutes. Probes were hybridized for 2 hours at 42°C followed by signal amplification. For fluorescent detection, the label probe sets for KIR2DL5 and CD45 were conjugated to Opal 570 and 690 nm (Akoyo Biosciences), respectively. Assays were typically performed in parallel with positive (ubiquitin C [UBC]) and negative (bacterial gene dapB) controls to assess both tissue RNA integrity and background signals. The slides were scanned by a 3DHistech P250 high-capacity slide scanner by 3 channels with filter settings for DAPI, FITC, and Cy7. Staining was analyzed with Volocity software by a trained researcher.

### IHC staining and imaging.

The same cohorts of TMAs used in RNAScope ISH were deparaffinized, followed by antigen retrieval with citrate unmasking buffer (CST) in a steamer for 20 minutes at a sub-boiling temperature (95°C–98°C). Slides were then blocked by 3% hydrogen peroxidase solution at RT for 10 minutes and subsequently by 10% normal goat serum at RT for 1 hour. A rabbit anti-PVR (clone D8A5G, CST) mAb was used at a dilution of 1:200 for overnight incubation at 4°C. The slides were then incubated with boost detection reagent (HRP, CST) at RT for 30 minutes, followed by SignalStain DAB (CST) and hematoxylin nuclear counterstaining. Positive and negative controls (FFPE cell blocks) were included in each staining.

### Xenograft models of human cancers.

For the subcutaneous A427 tumor model, 6- to 8-week-old NSG or NSG–hIL-15 mice were inoculated s.c. with 3 × 10^6^ A427 cells on the hind flanks. Three or five days later, mice were randomized into 2 groups (*n* = 6 or 8) and treated with KIR2DL5^+^ primary NK cells (1 × 10^7^) and 200 μg anti-KIR2DL5 mAb (clone F8B30) or isotype control (mIgG1) intratumorally twice (once every 3 days). Tumors were measured by caliper, and tumor volume was calculated as (width^2^ × length)/2.

For the intravenous A427 tumor model, NSG mice were injected intravenously (i.v.) with 1 × 10^6^ luciferase-expressing A427 cells (A427-luc2). One day later, mice underwent bioluminescence imaging (BLI) and were allocated to 2 groups (*n* = 5) based on similar average photon flux (photons/second). Mice were then treated i.v. with KIR2DL5^+^ primary NK cells (1 × 10^7^) and 200 μg F8B30 or mIgG1 twice (once every 3 days). Lung tumor growth was monitored by BLI weekly, and mice were euthanized when the total flux reached to 1 × 10^8^ photons/second.

For the intravenous Jurkat tumor model, NSG mice were injected i.v. with 5 × 10^5^ luciferase-expressing Jurkat cells (Jurkat-luc2). Four days later, mice were allocated to 2 groups (*n* = 4 or 6) based on similar average photon flux (photons/second), and treated i.v. with KIR2DL5^+^ primary NK cells (1 × 10^7^) and 200 μg F8B30 or mIgG1 twice (once every 3 days). Tumor growth was monitored by BLI, and mice were euthanized when the total flux reached to 1 × 10^10^ photons/second. For all BLI, d-luciferin (150 mg/kg; Gold Biotechnology) was administered by intraperitoneal injection to mice for 10 minutes before imaging. The data were analyzed with Living Image 3.0 software.

### Data availability.

Previously published Gene Expression Omnibus (GEO) data that were reanalyzed here are available under accession codes GSE7904, GSE19069, and GSE39612.

### Statistics.

Statistical analyses were performed in GraphPad Prism, version 9.0 (GraphPad Software) using appropriate tests as indicated in the figure legends (unpaired 2-tailed *t* test, paired 2-tailed *t* test, 1-way ANOVA followed by Tukey’s or Dunnett’s multiple-comparison test, 2-way ANOVA followed by Šidák’s multiple-comparison test, multiple *t* test, and log-rank test for Kaplan-Meier survival curves). The data are expressed as mean ± SEM of *n* = 3 or more determinations. A *P* value of less than 0.05 was considered statistically significant.

### Study approval.

All mouse protocols were approved by the Animal Care and Use Committee of Albert Einstein College of Medicine in accordance with the NIH guidelines for the care and use of laboratory animals.

## Author contributions

XR, MP, and X Zang designed the study. XR, MP, PX, YW, and DC performed research. XR performed animal studies. MP, PX, and YW generated in-house anti-KIR2DL5 mAbs. PMG and DZ performed bioinformatics analysis. HW, YS, XD, QS, YL, X Zhang, and WE contributed critical reagents and provided experimental support. XR wrote the manuscript. XR and MP prepared figures. XR, MP, and DC revised the manuscript. X Zang and MP reviewed and edited the manuscript. X Zang supervised and acquired funding for the study. As XR conducted 60% of the in vitro study and 90% of the in vivo study, wrote the manuscript, and revised the manuscript, XR is listed as the first co–first author.

## Supplementary Material

Supplemental data

## Figures and Tables

**Table 2:**
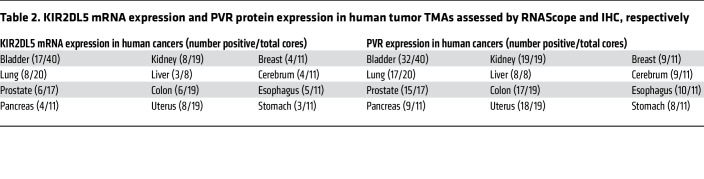
KIR2DL5 mRNA expression and PVR protein expression in human tumor TMAs assessed by RNAScope and IHC, respectively

**Table 1:**
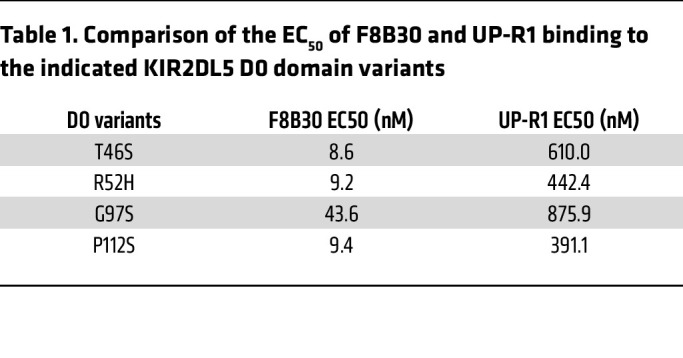
Comparison of the EC_50_ of F8B30 and UP-R1 binding to the indicated KIR2DL5 D0 domain variants

## Version 1 11/15/2022

Electronic publication
