# Supplementary material for: Blockade of the immunosuppressive KIR2DL5/PVR pathway elicits potent human NK cell–mediated antitumor immunity
Source: J Clin Invest. 2022 Nov 15;132(22):e163620. doi: 10.1172/JCI163620 (PMC9663162; doi:10.1172/JCI163620)
Supplement: Supplemental data [file jci-132-163620-s028.pdf]

Supplemental Materials for

Blockade of the immunosuppressive KIR2DL5-PVR pathway elicits potent human NK cell-mediated anti-tumor immunity

Xiaoxin Ren[1,‡], Mou Peng[1,§], Peng Xing[1], Yao Wei[1], Phillip M. Galbo Jr.[1,2], Devin Corrigan[1], Hao Wang[1], Yingzhen Su[1], Xiaoshen Dong[1], Qizhe Sun[1], Yixian Li[1,3], Xiaoyu Zhang[1], Winfried Edelmann[4], Deyou Zheng[2,5], Xingxing Zang[1,6,7,8]

[1]Department of Microbiology & Immunology, Albert Einstein College of Medicine, Bronx, NY, USA

[2]Department of Genetics, Albert Einstein College of Medicine, Bronx, NY, USA.

[3]Division of Pediatric Hematology/Oncology/Transplant and Cellular Therapy, Children's Hospital at Montefiore, Bronx, NY, USA

[4]Departments of Cell Biology, Albert Einstein College of Medicine, Bronx, NY, USA

[5]Departments of Neurology and Neuroscience, Albert Einstein College of Medicine, Bronx, NY, USA

[6]Department of Oncology, Albert Einstein College of Medicine, Bronx, NY, USA

[7]Department of Medicine, Albert Einstein College of Medicine, Bronx, NY, USA

[8]Department of Urology, Albert Einstein College of Medicine, Bronx, NY, USA

The PDF file includes:

Supplemental Figure 1-8

Supplemental Table 1

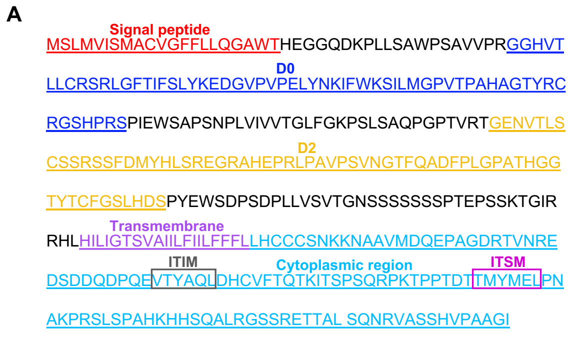

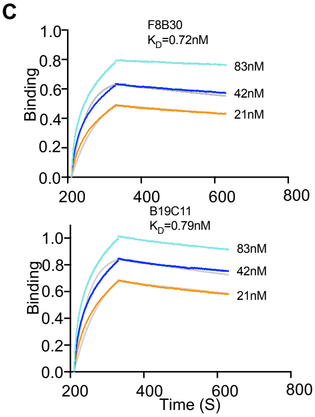

D

Clone	KD (nM)
F8B30	0.72± 0.023
B19C11	0.79±0.020
B2A18	0.104±0.003
B7B23	0.04±0.003
B11B4	0.25± 0.011
B33C12	0.36± 0.012
E12B11	0.14± 0.006
G11B22	0.42± 0.006

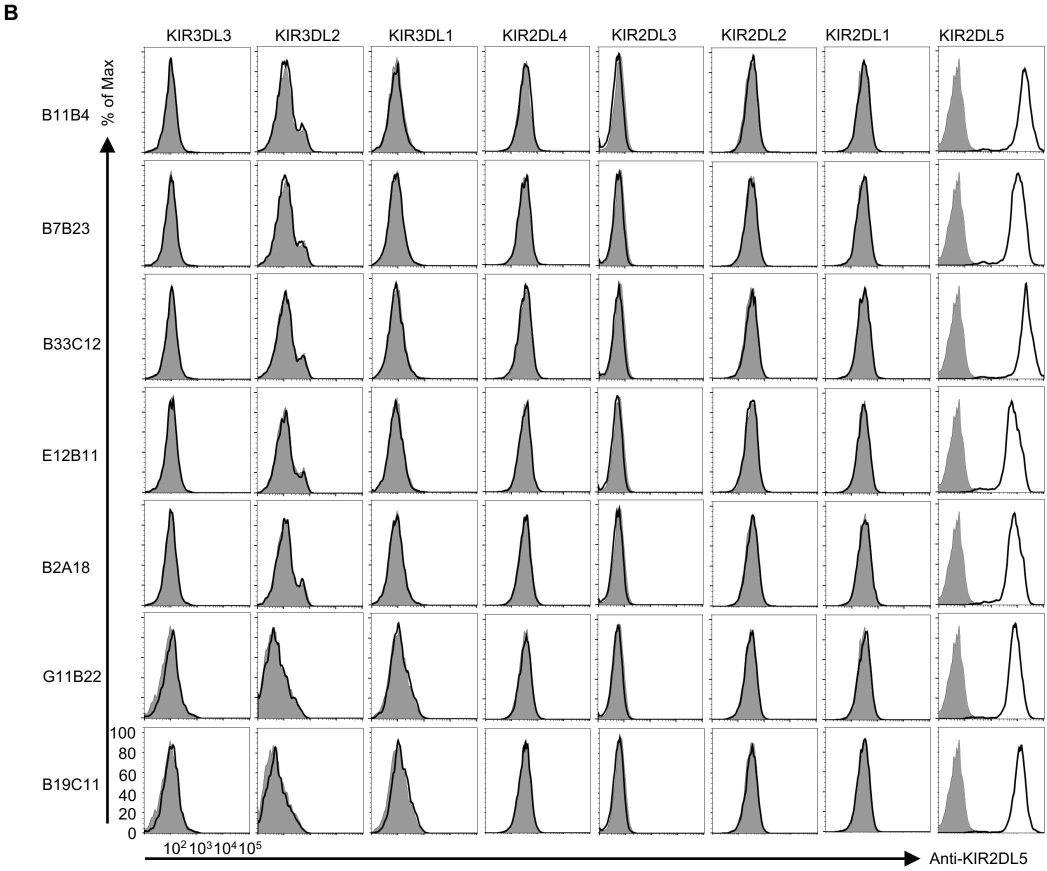

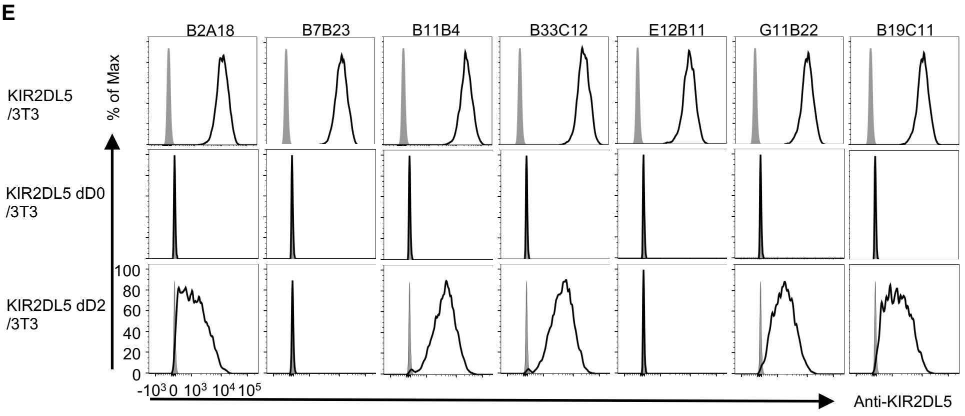

Supplemental Figure 1. Characterization of anti-KIR2DL5 specific monoclonal antibodies. (Related to Figure 1).

(A) The protein sequence of KIR2DL5. The extracellular domain of KIR2DL5 was composed of tandem D0-D2 domains. The cytoplasmic tail of KIR2DL5 contained an immunoreceptor tyrosine-based inhibition motif (ITIM) and immunoreceptor tyrosine-based switch motif (ITSM). Domains were predicted and annotated based on UniProtKB (Q8N109).

(B) The specificity of anti-KIR2DL5 mAbs. 3T3 cells transduced with indicated KIR family members were stained with 5 μg/ml of indicated anti-KIR2DL5 mAbs (open) or mIgG1 (shaded).

(**C, D)** The affinity of anti-KIR2DL5 mAbs. **C**: Kinetic binding curves for F8B30 and B19C11. **D**: Data were acquired from kinetic binding curves detected by the Octet Red96 BLI instrument for indicated clones.

(E) 3T3 cells transduced with D0-deleted (KIR2DL5 dD0) or D2-deleted KIR2DL5 (KIR2DL5 dD2) were stained with 5 μg/ml of indicated anti-KIR2DL5 mAbs.

In **B-E**, data are representative of three independent experiments.

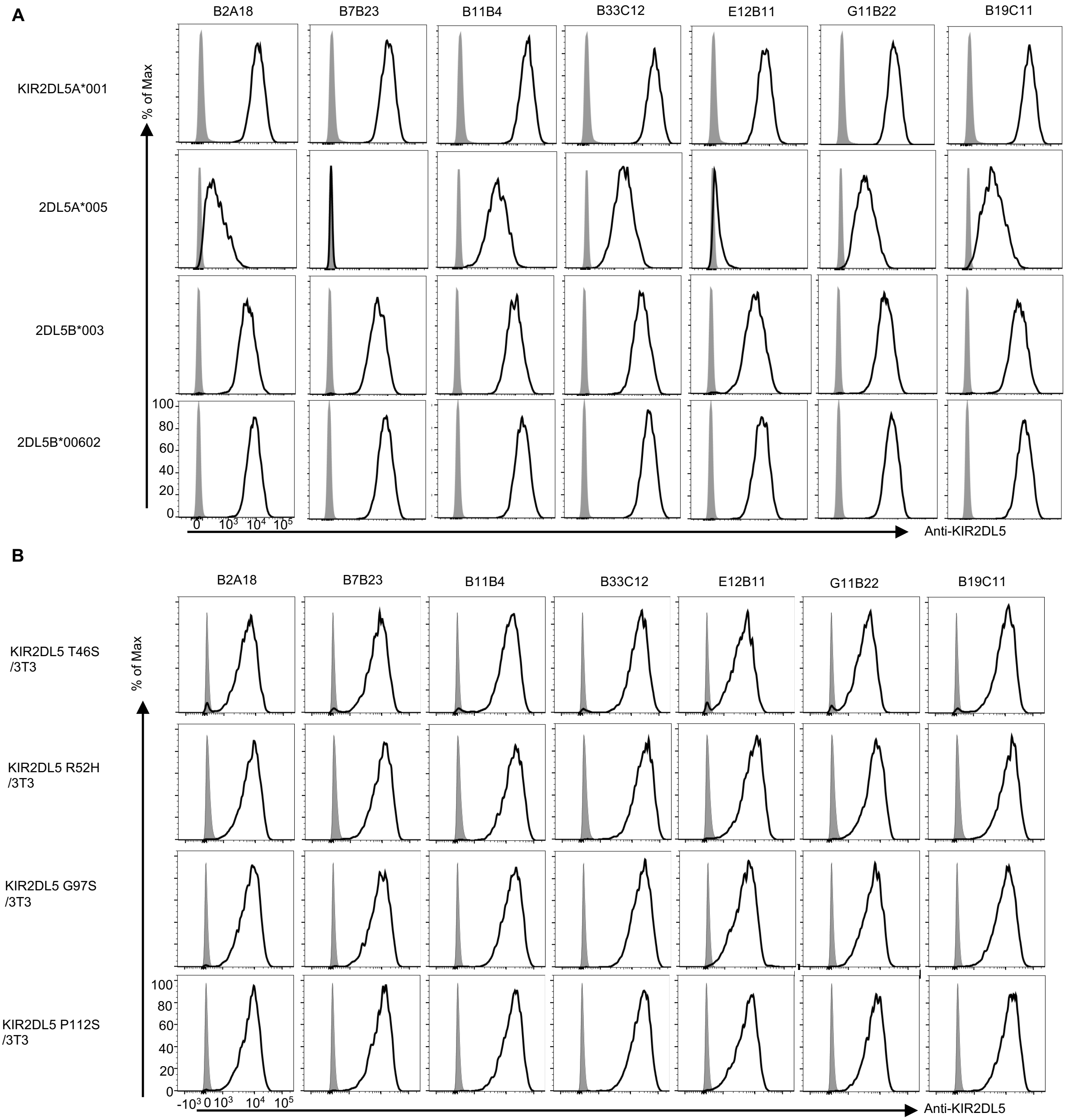

Supplemental Figure 2. Allelic polymorphism affected mAb recognition of KIR2DL5. (Related to Figure 1).

(A) The binding of anti-KIR2DL5 mAbs (5 μg/ml, open) and mIgG1 (shaded) to the 3T3 cells expressing different KIR2DL5A and KIR2DL5B alleles.

(B) The binding of anti-KIR2DL5 mAbs (5 μg/ml, open) and mIgG1 (shaded) to the 3T3 cells expressing different KIR2DL5 D0 variants.

In **A** and **B**, data are representative of two independent experiments.

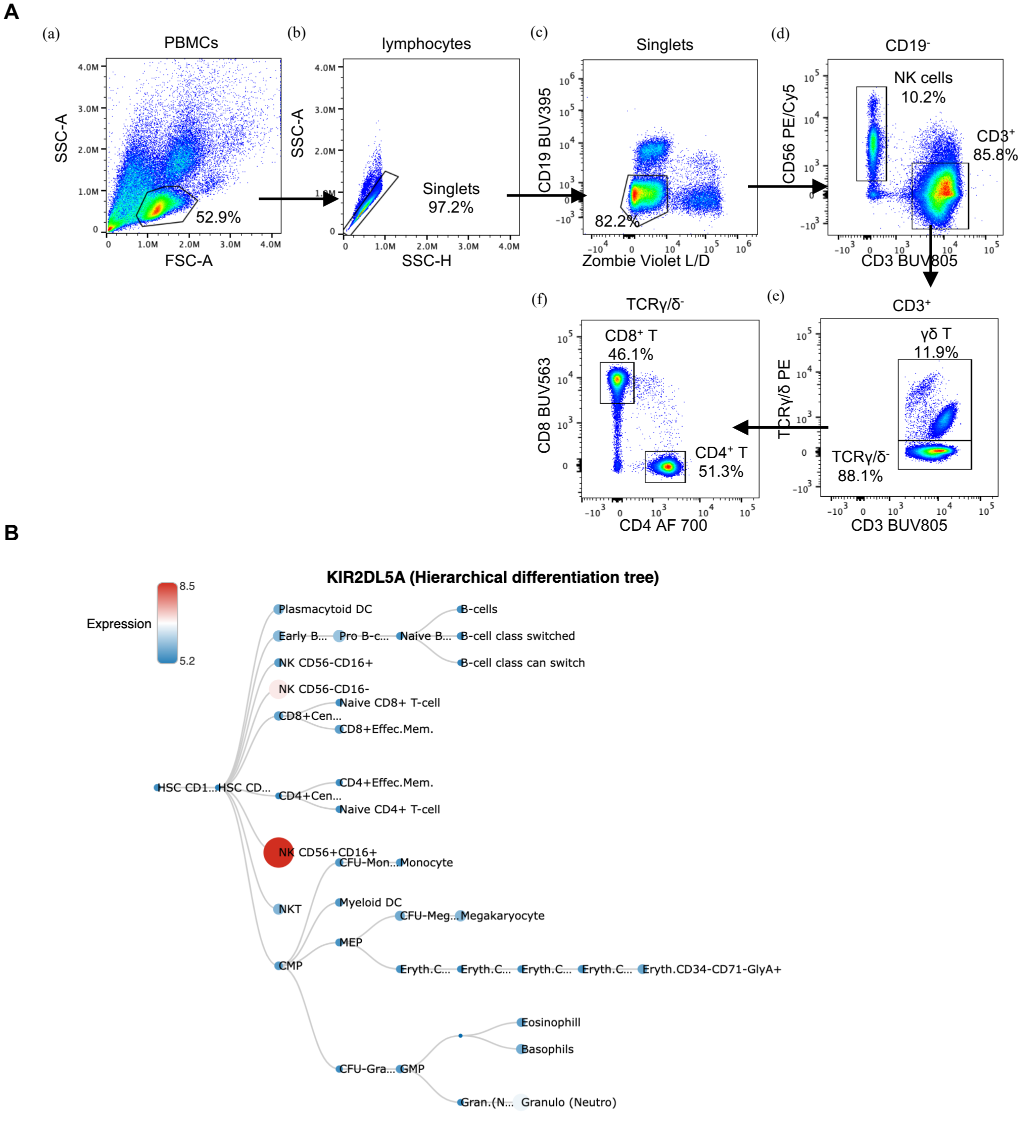

Supplemental Figure 3. KIR2DL5 was predominantly expressed on mature NK cells. (Related to Figure 2).

(A) Gating strategy for immune cell subsets in human PBMCs. (a) The major lymphocytes was gated based on the FCS-A and SSC-A; (b) Doublets were excluded based on plotted in SSC-H/SSC-A. (c) Live CD19$^-$ cells were gated from single cells based on CD19 and Live/Dead blue staining; (d) CD3$^-$ CD56$^+$ cells were defined as NK cells; (e) From CD3$^+$ CD56$^-$ cells, γδ T were defined based on TCRγ/δ staining; (f) CD3$^+$ TCRγ/δ$^-$ cells were then divided into CD4$^+$ T and CD8$^+$ T subsets.

(B) *KIR2DL5A* expression in human normal hematopoietic cells. Hierarchical differentiation tree was generated from BloodSpot database (https://servers.binf.ku.dk/bloodspot/?gene=KIR2DL5A&dataset=DMAP).

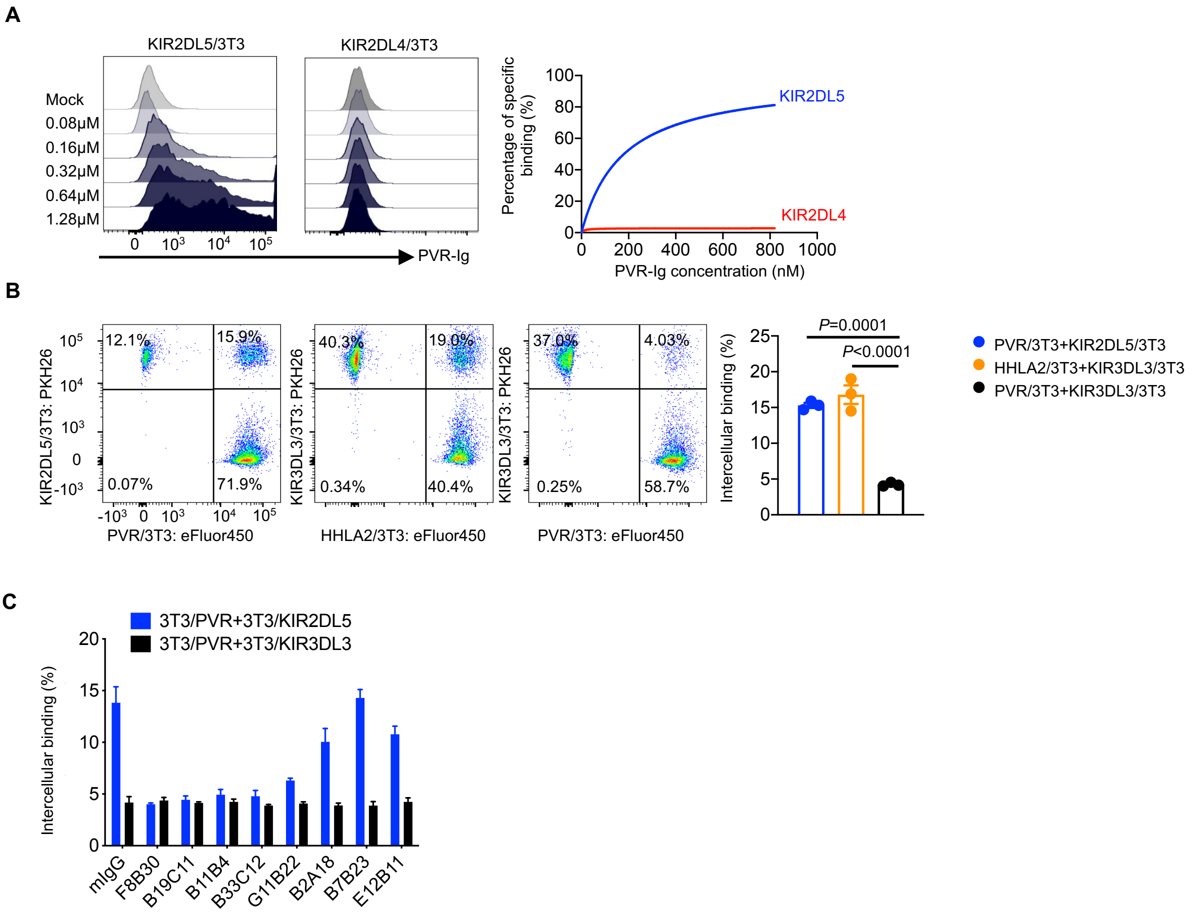

Supplemental Figure 4. Characterization of KIR2DL5 as a binding partner for PVR. (Related to Figure 3)

(A) Flow cytometric analysis of PVR binding to KIR2DL5/3T3 or KIR2DL4/3T3 at increasing concentrations of PVR-Ig.

(B) KIR2DL5-PVR interaction by intercellular conjugate assay. Left: Prelabeled KIR2DL5/3T3 and PVR/3T3 cells were co-incubated and then analyzed by flow cytometry. KIR3DL3/3T3 + HHLA2/3T3 and KIR3DL3/3T3 + PVR/3T3 co-incubation were used as a positive and negative control, respectively. Right: Summary of the intercellular conjugation of indicated groups. Data are mean ± SEM from three independent experiments. P values by a one-way ANOVA.

(C) Intercellular conjugate assay between KIR2DL5/3T3 and PVR/3T3 in the presence of indicated anti-KIR2LD5 mAbs. KIR3DL3/3T3 + PVR/3T3 co-incubation was used as a negative control.

In **A** and **C**, data are representative of three independent experiments.

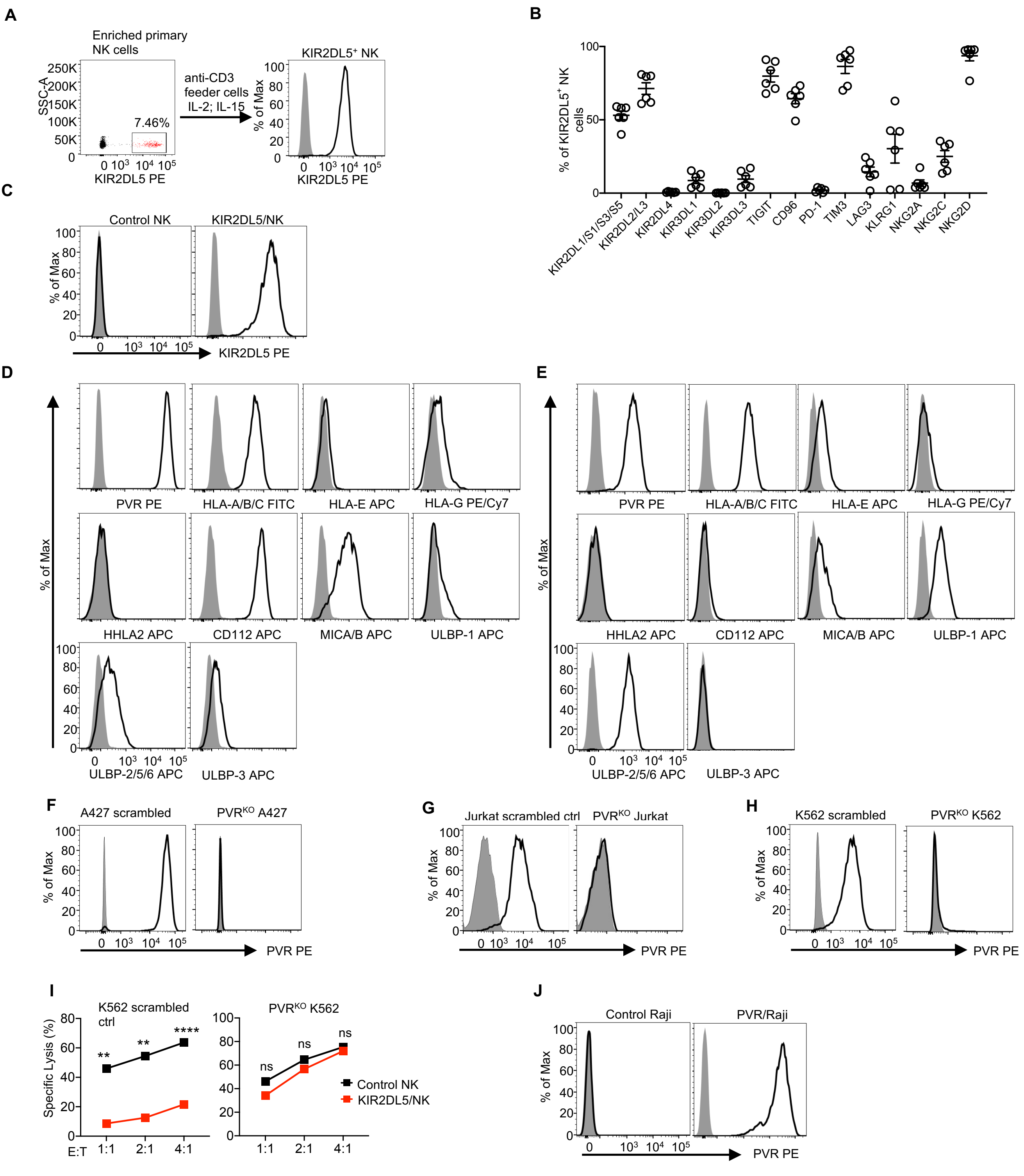

A
Enriched primary NK cells
SSC-A
7.46%
KIR2DL5 PE
anti-CD3 feeder cells IL-2; IL-15
KIR2DL5⁺ NK
% of Max
B
% of KIR2DL5⁺ NK cells
KIR2DL1/S1/S3/S5
KIR2DL2/L3
KIR2DL4
KIR3DL1
KIR3DL2
KIR3DL3
TIGIT
CD96
PD-1
TIM3
LAG3
KLRG1
NKG2A
NKG2C
NKG2D
C
Control NK
KIR2DL5/NK
D
E
PVR PE
HLA-A/B/C FITC
HLA-E APC
HLA-G PE/Cy7
HHLA2 APC
CD112 APC
MICA/B APC
ULBP-1 APC
ULBP-2/5/6 APC
ULBP-3 APC
F
A427 scrambled
PVRKO A427
G
Jurkat scrambled ctrl
PVRKO Jurkat
H
K562 scrambled
PVRKO K562
I
K562 scrambled ctrl
PVRKO K562
Specific Lysis (%)
E:T
Control NK
KIR2DL5/NK
J
Control Raji
PVR/Raji

Supplemental Figure 5. KIR2DL5 mediated PVR$^+$ tumor immune resistance to NK cell cytotoxicity. (Related to Figure 4).

(A) KIR2DL5$^+$ primary NK cells were sorted from human PBMCs and cultured *in vitro*. The expression of KIR2DL5 was confirmed with F8B30 (open) or mIgG1 (shaded) by flow cytometry.

(B) Expression of other immune receptors on KIR2DL5$^+$ primary NK cells in **A**. Data are represented as means ± SEM of six different donors.

(C) Primary NK cells were transduced with empty vector (control NK) or KIR2DL5 (KIR2DL5/NK) and examined for KIR2DL5 expression with F8B30 (open) or mIgG1 (shaded).

(D,E) The expression of activating or inhibitory ligands on A427 (**D**) and Jurkat (**E**). Cells was stained by the indicated markers (open) and isotype control (shaded).

(F-H) Scrambled control and PVR^{KO} A427 (**F**), Jurkat (**G**) and K562 (**H**) cell lines were generated and examined for PVR expression with anti-PVR mAb (open) or isotype control (shaded).

(I) Lysis of scrambled control or PVR^{KO} K562 cells by KIR2DL5$^+$ primary NK cells or control KIR2DL5$^-$ NK cells at indicated E:T ratios. Data are mean for duplicate measurements and representative of three independent experiments with three different donors.

(J) Control Raji and PVR/Raji cell lines were generated and examined for PVR expression with anti-PVR mAb (open) or isotype control (shaded).

In **A, C-H** and **J**, data are representative of three independent experiments. P values by a multiple unpaired t-test (**I**). **P < 0.01, ****P < 0.0001; ns, not significant.

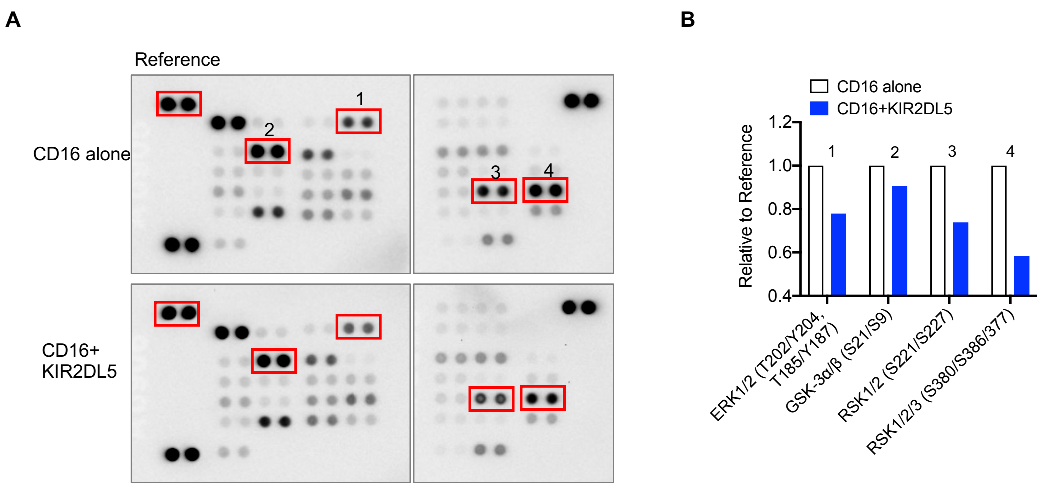

Supplemental Figure 6. ERK1/2/p90RSK pathway was involved in KIR2DL5 downstream signaling. (Related to Figure 5).

(A, B) A human phospho-kinase array of KIR2DL5^{+} primary NK cells after crosslinking with anti-CD16 and mIgG1 (CD16 alone), or anti-KIR2DL5 mAb F8B30 (CD16+KIR2DL5) for 2 minutes. **A**: Kinase spots with significantly different densities between two groups are indicated. **B**: Relative quantification of the phosphorylation level of indicated kinases. Data are representative of two independent experiments.

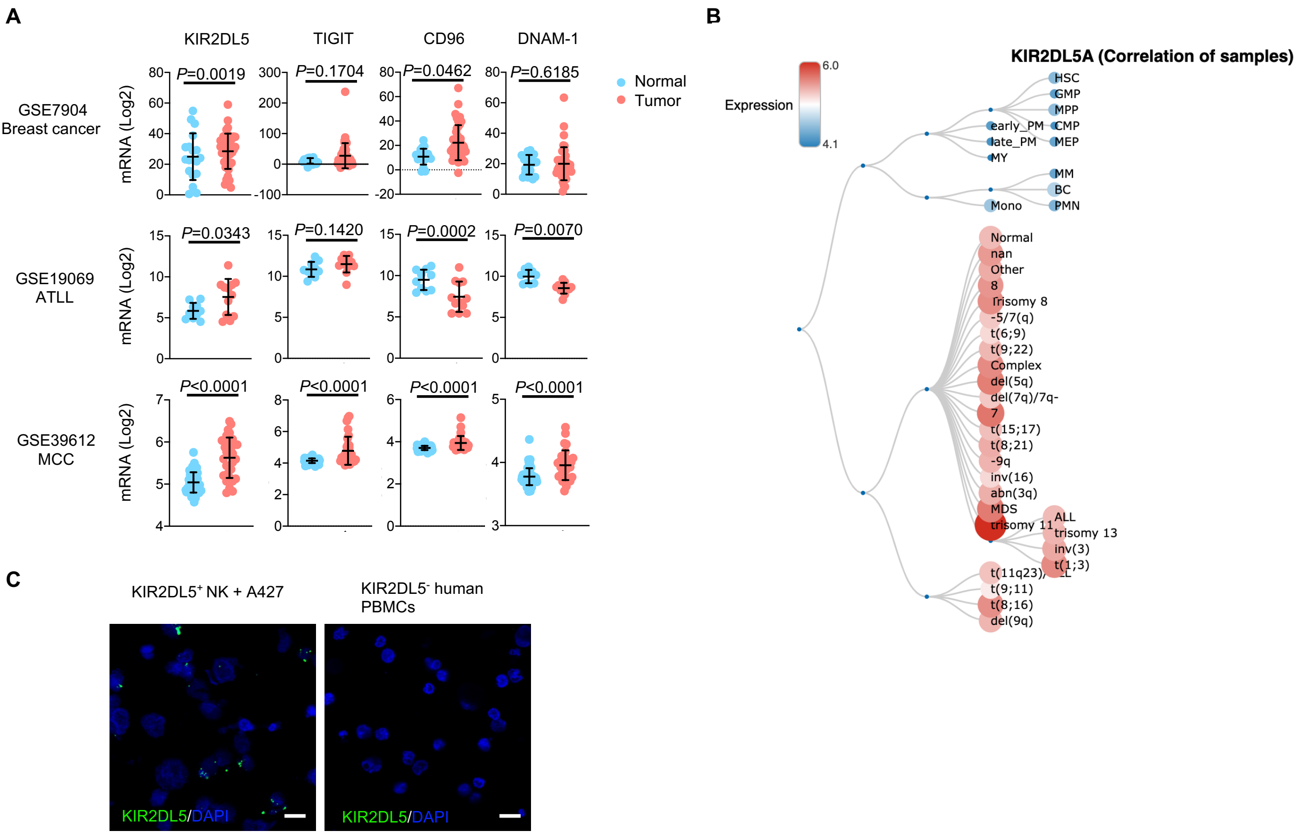

Supplemental Figure 7. KIR2DL5 was upregulated in solid and hematopoietic tumors. (Related to Figure 6).

(A) The mRNA expression of KIR2DL5, TIGIT, CD96, and DNAM-1 in human tumors versus corresponding normal tissues by analyzing indicated Gene Expression Omnibus (GEO) databases. (Breast cancer, n = 43 versus 7; ATLL (adult T cell leukemia/lymphoma, n = 12 versus 10 ; MCC (merkel cell carcinoma), n = 27 versus 64 samples of human tumor versus normal tissues). Data are mean ± SEM.

(B) Analysis of KIR2DL5A mRNA expression in primary human AML (acute myeloid leukemia) across cytogenetic subtypes in comparison with normal hematopoietic cells. Hierarchical differentiation tree was generated from BloodSpot database (https://servers.binf.ku.dk/bloodspot/?gene=KIR2DL5A&dataset=MERGED_AML)

(C) Representative RNAScope images of positive staining for KIR2DL5 with KIR2DL5^{+} primary NK mixed with A427 cell slide and negative staining with KIR2DL5^{-} human PBMCs slide. Scale bar, 10 μm.

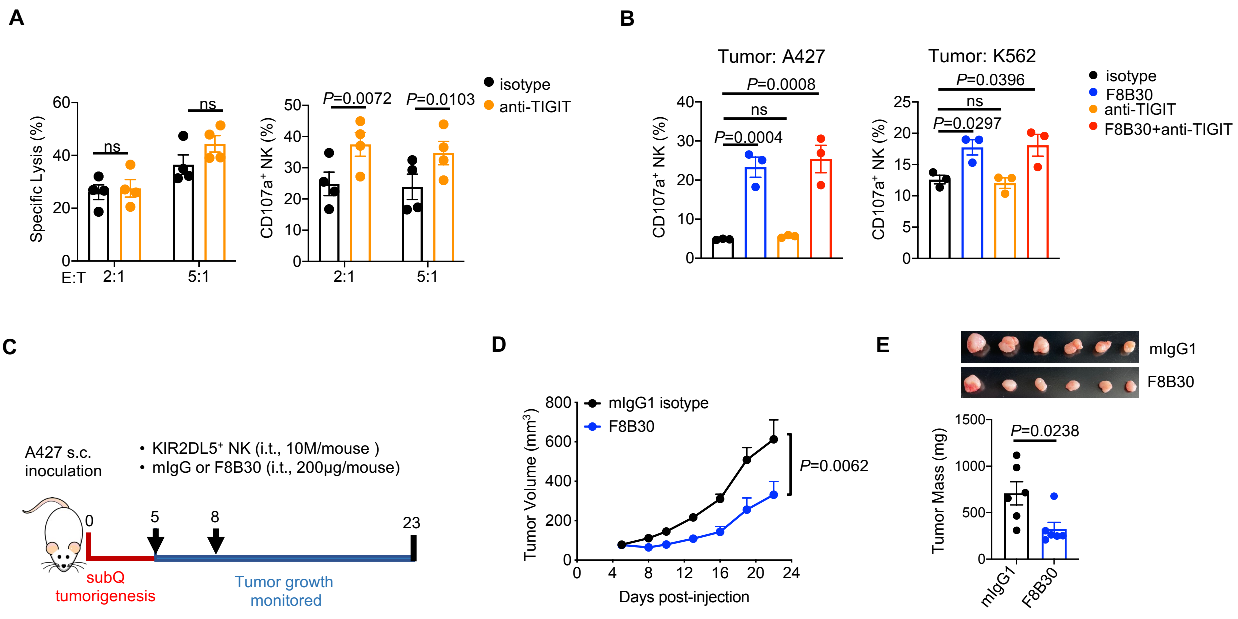

Supplemental Figure 8. KIR2DL5 blockade promoted NK-based anti-tumor immunity. (Related to Figure 7).

(A) Tumor lysis and NK degranulation after co-culturing IL-2+ IL-15 stimulated primary NK cells with A427 cells in the presence of anti-TIGIT mAb or isotype control.

(B) KIR2DL5^{+} primary NK cell degranulation after co-culturing with A427 or K562 cells in the presence of indicated mAbs at indicated E:T= 2:1.

(C-E) A427 subcutaneous tumor model in NSG-hIL15 mice. (**C**) Schematic of experimental design. NSG-hIL-15 mice were engrafted s.c. with A427 (3×10^{6}/mouse) on day 0, followed by randomization on day 5 and i.t. treatment with KIR2DL5^{+} primary NK cells together with mIgG1 or F8B30 every three days for twice. (**D**) Growth of A427 tumors. n=6 tumors per group. (**E**) Tumor mass and images of each group at day 23.

In **A** and **B**, data are means ± SEM of three independent experiments with three or four different donors. P values by a two-tailed unpaired Student's *t*-test (**A, E**), one-way ANOVA (**B**), two-way ANOVA (**D**). s.c., subcutaneously; i.t., intratumorally. ns, not significant.

Supplemental Table 1. Antibodies used in this study

Antibodies	Source	Identifier
Anti-human IgG Fc APC (clone HP6017)	Biolegend	Cat# 409306; RRID:AB_11150591
Anti-human IgG Fc PE (clone HP6017)	Biolegend	Cat# 409303 RRID:AB 10900424
F(ab')2-goat anti-mouse IgG APC (polyclonal antibody)	eBiocience	Cat# 17-4010-82; RRID:AB_2573203
Goat anti-mouse IgG PE (polyclonal antibody)	Biolegend	Cat# 405307; RRID:AB_315010
Mouse IgG1 isotype (clone HKSP)	Leinco Techologies	Cat# I-536; RRID:AB_2737545
Anti-human CD3-BUV805 (clone UCHT1)	BD	Cat# 612895; RRID:AB_ 2739277
Anti-human CD4-Alexa 700 (clone RPA-T4)	Biolegend	Cat# 300526; RRID:AB_493743
Anti-human CD8-BUV563 (clone RPA-T8)	BD	Cat# 612914; RRID:AB_2744461
Anti-human CD56-PE/Cy5 (clone 5.1H11)	Biolegend	Cat# 362516; RRID:AB_2564089
Anti-human CD57-BV510 (clone QA17A04)	Biolegend	Cat# 393313; RRID:AB_2750341
Anti-human TCR γδ-PE (clone B1)	Biolegend	Cat# 331210; RRID:AB_1089218
Anti-human CCR7-BV750 (clone G043H7)	Biolegend	Cat# 353253; RRID:AB_2800944
Anti-human CD45RA-BV570 (clone HI100)	Biolegend	Cat# 304131; RRID:AB_10897946
Anti-human PVR-PE (clone SKII.4)	Biolegend	Cat# 337609; RRID:AB_2253258
Anti-human DNAM-1-FITC (clone 11A8)	Biolegend	Cat# 338303; RRID:AB_1279145
Anti-human TIGIT-APC (clone A15153G)	Biolegend	Cat# 372705; RRID:AB_2632731
Anti-human CD96-PerCP/Cy5.5 (clone NK92.39)	Biolegend	Cat# 338411; RRID:AB_2566143
Anti-human CD16-BUV496 (clone 3G8)	BD	Cat# 612945; RRID:AB_2744294
Anti-human CD19-BUV395 (clone SJ25C1)	BD	Cat# 563551; RRID:AB_2738272
Anti-human KLRG1-APC (clone SA231A2)	Biolegend	Cat# 367716; RRID:AB_2572161
Anti-human NKp46-Alexa 647 (clone 9E2)	Biolegend	Cat# 331909; RRID:AB_1027674

Anti-human KIR3DL2-Alexa 700 (clone 539304)	R&D	Cat# FAB2878N025
Anti-human KIR3DL3-PE (clone 26E10)	Zang lab	Wei, et al. 2021
Anti-human NKG2D-APC/Cy7 (clone 1D11)	Biolegend	Cat# 320824; RRID:AB_2566660
Anti-human KIR2DL1/S1/S3/S5-PE/Cy7 (clone HP-MA4)	Biolegend	Cat# 339511; RRID:AB_2565578
Anti-human NKG2C-Alexa 488 (clone 134591)	R&D	Cat# FAB138G025; RRID:AB_10890779
Anti-human KIR2DL2/L3-PerCP/Cy5.5 (clone DX27)	Biolegend	Cat# 312613; RRID:AB_2564334
Anti-human KIR2DL4-APC (clone mAb 33)	Biolegend	Cat# 347007; RRID:AB_2249479
Anti-human 2B4-BV605 (clone C1.7)	Biolegend	Cat# 329535; RRID:AB_2814197
Anti-human NKG2A-BV650 (clone 131411)	BD	Cat# 747920; RRID:AB_2872381
Anti-human NKp44-BV711 (clone p44-8)	BD	Cat# 744303; RRID:AB_2742133
Anti-human NKp30-BV786 (clone p30-15)	BD	Cat# 743172; RRID:AB_2741323
Anti-human CD107a-Alexa 488 (clone H4A3)	Biolegend	Cat# 328610; RRID:AB_1227504
Anti-human IFN-γ-PerCP/Cy5.5 (clone B27)	Biolegend	Cat# 506528; RRID:AB_2566187
Anti-human TNF-α-PE/Cy7 (clone mab11)	Biolegend	Cat# 502930; RRID:AB_2204079
Anti-human KIR2DL5-PE (clone UP-R1)	Biolegend	Cat# 341303; RRID:AB_1595545
Anti-human KIR2DL5-PE (clone F8B10)	This paper	N/A
Anti-human KIR2DL5 (clone F8B30)	This paper	N/A
Anti-human KIR2DL5 (clone B7B23)	This paper	N/A
Anti-human KIR2DL5 (clone B33C12)	This paper	N/A
Anti-human KIR2DL5 (clone E12B11)	This paper	N/A
Anti-human KIR2DL5 (clone B2A18)	This paper	N/A
Anti-human KIR2DL5 (clone G11B22)	This paper	N/A
Anti-human KIR2DL5 (clone B19C11)	This paper	N/A
Anti-human KIR2DL5 (clone B11B4)	This paper	N/A
Purified anti-human CD3 antibody (clone OKT3)	Biolegend	Cat# 317326; RRID:AB_11150592

Purified anti-human CD56 (clone 5.1H11)	Biolegend	Cat# 362502; RRID:AB_2563558
Purified anti-human CD16 antibody (clone 3G8)	Biolegend	Cat# 302014; RRID:AB_314214
Purified anti-human DNAM-1 antibody (clone DX11)	BD	Cat# 559787; RRID:AB_397328
Purified anti-human TIGIT antibody (clone MBSA43)	eBiocience	Cat# 16-9500-82; RRID:AB_10718831
Anti-PVR (clone D8A5G)	Cell Signaling Technology	Cat# 81254S; RRID:AB_2799970
Anti-phosphotyrosine antibody (clone 4G10)	Merck Millipore	Cat# 05321; RRID:AB_309678
Anti-β-actin (clone C11)	Santa Cruz	Cat# sc-1615; RRID:AB_630835
Anti-phospho ERK1/2 (Thr202/Tyr204) (clone 6B8B69)	Biolegend	Cat# 369502; RRID:AB_2721735
Anti-total ERK1/2 (clone 137F5)	Cell Signaling Technology	Cat# 4695T; RRID:AB_2339400
Anti-phospho Vav1 (Tyr160) (polyclonal antibody)	Invitrogen	Cat# 44-482; RRID:AB_2533661
Anti-total Vav1 (clone D45G3)	Cell Signaling Technology	Cat# 4657S; RRID:AB_10624865
Anti-phospho-p90RSK (Thr359/Ser363) (polyclonal antibody)	Cell Signaling Technology	Cat# 9344S; RRID:AB_915783
Anti-total p90RSK (clone 32D7)	Cell Signaling Technology	Cat# 9355S;
Anti-SHP-1 (clone C14H6)	Cell Signaling Technology	Cat# 3759; RRID:AB_2173694
Anti-SHP-2 (clone D50F2)	Cell Signaling Technology	Cat# 3397; RRID:AB_2174959
Anti-phospho NF-κB p65 (Ser536) (clone 93H1)	Cell Signaling Technology	Cat# 3033S; RRID:AB_331284
Goat anti-rabbit IgG-HRP	Cell Signaling Technology	Cat# 7074S; RRID:AB_2099233
Goat anti-mouse IgG-HRP	Jackson ImmunoResearch	Cat# 115-035-003; RRID:AB_10015289
Rabbit anti-goat IgG-HRP	Jackson ImmunoResearch	Cat# 305-035-003; RRID:AB_2339400
